# Direct interaction of TrkA/CD44v3 is essential for NGF-promoted aggressiveness of breast cancer cells

**DOI:** 10.1186/s13046-022-02314-4

**Published:** 2022-03-28

**Authors:** Sarah Trouvilliez, Julien Cicero, Romain Lévêque, Léo Aubert, Cyril Corbet, Alexandre Van Outryve, Karolin Streule, Pierre-Olivier Angrand, Pamela Völkel, Romain Magnez, Guillaume Brysbaert, Caroline Mysiorek, Fabien Gosselet, Roland Bourette, Eric Adriaenssens, Xavier Thuru, Chann Lagadec, Jérôme de Ruyck, Véronique Orian-Rousseau, Xuefen Le Bourhis, Robert-Alain Toillon

**Affiliations:** 1 University Lille, CNRS, INSERM, CHU Lille, UMR9020-U1277 - CANTHER - Cancer Heterogeneity Plasticity and Resistance to Therapies, F-59000 Lille, France; 2 University Artois, UR 2465, Laboratoire de la Barrière Hémato-Encéphalique (LBHE), F-62300 Lens, France; 3 Karlsruhe Institute of Technology, Institute of Toxicology and Genetics, 76344 Eggenstein-Leopoldshafen, Germany; 4 University Lille, CNRS, UMR 8576 - UGSF - Unité de Glycobiologie Structurale et Fonctionnelle, F-59000 Lille, France; 5 Université de Lille, Faculté des Sciences et Technologies, UMR CNRS 9020- INSERM U1277 - CANTHER - Cancer Heterogeneity Plasticity and Resistance to Therapies, Bâtiment SN3, 3ème étage, Cité scientifique, 59655 Villeneuve d’Ascq, France

**Keywords:** CD44v3, TrkA, NGF, Breast cancer, Metastasis

## Abstract

**Background:**

CD44 is a multifunctional membrane glycoprotein. Through its heparan sulfate chain, CD44 presents growth factors to their receptors. We have shown that CD44 and Tropomyosin kinase A (TrkA) form a complex following nerve growth factor (NGF) induction. Our study aimed to understand how CD44 and TrkA interact and the consequences of inhibiting this interaction regarding the pro-tumoral effect of NGF in breast cancer.

**Methods:**

After determining which CD44 isoforms (variants) are involved in forming the TrkA/CD44 complex using proximity ligation assays, we investigated the molecular determinants of this interaction. By molecular modeling, we isolated the amino acids involved and confirmed their involvement using mutations. A CD44v3 mimetic peptide was then synthesized to block the TrkA/CD44v3 interaction. The effects of this peptide on the growth, migration and invasion of xenografted triple-negative breast cancer cells were assessed. Finally, we investigated the correlations between the expression of the TrkA/CD44v3 complex in tumors and histo-pronostic parameters.

**Results:**

We demonstrated that isoform v3 (CD44v3), but not v6, binds to TrkA in response to NGF stimulation. The final 10 amino acids of exon v3 and the TrkA H112 residue are necessary for the association of CD44v3 with TrkA. Functionally, the CD44v3 mimetic peptide impairs not only NGF-induced RhoA activation, clonogenicity, and migration/invasion of breast cancer cells in vitro but also tumor growth and metastasis in a xenograft mouse model. We also detected TrkA/CD44v3 only in cancerous cells, not in normal adjacent tissues.

**Conclusion:**

Collectively, our results suggest that blocking the CD44v3/TrkA interaction can be a new therapeutic option for triple-negative breast cancers.

**Supplementary Information:**

The online version contains supplementary material available at 10.1186/s13046-022-02314-4.

## Background

Breast cancer is the most prevalent cancer in women worldwide. With a slight increase in annual incidence, it is estimated that 1 in 8 women will be affected in her lifetime. Despite significant progress in both patient care and treatments, more than 684,000 women died of breast cancer in 2020 [1]. These deaths are largely attributable to metastatic progression [2]. Among the main breast cancer subtypes (luminal, Her2-positive and triple-negative (TN) subtypes), the TN subtype is the most aggressive. Metastasis of TN breast cancer (TNBC) is associated with an overall survival 3.5-fold lower than that of luminal breast cancer [3]. This low overall survival is probably related to a higher metastasis rate. As Paget described, breast cancer metastasis is not a random mechanism [4] as described by the seed and soil theory; it involves crosstalk between tumor cells and microenvironmental factors such as the extracellular matrix and growth factors. Among these factors, hyaluronan, a major component of the extracellular matrix, binds to the CD44 glycoprotein, which is the main hyaluronan receptor. CD44 is involved in various physiological functions, including cell proliferation, migration, invasion, and the immune response. Since this discovery, many studies have underlined the importance and expression of CD44 in different cancers [5]. The diversity of CD44 functions is due to the multiplicity of CD44 ligands (e.g., hyaluronan and osteopontin), posttranslational modifications (such as glycosylation) or isoform expression. Of the 20 CD44 gene exons, 12 are variable splicing exons, allowing the formation of more than 700 isoforms. However, not all combinations are expressed [6], and at least 20 CD44 isoforms have been described to date. These variants exhibit specific functions and pattern expression [7]. The most abundant isoform is the CD44 standard transcript, but several variants of CD44 have been shown to be involved in CD44 responses. In cancers, two specific alternative splicing variants of CD44, variants 3 (CD44v3) and 6 (CD44v6), containing variable exons 8 and 11, respectively, interact with numerous tyrosine kinase receptors [8, 9]. Hence, the discovery of the differential expression of CD44 variants in cancer biology explained the role of variant 6 in the metastatic processes of rat tumors [10]. CD44v6 is a coreceptor in MET activation by MET ligands HGF/SF [11]. Furthermore, we recently emphasized that cells express different CD44 variants, including CD44v6, to orchestrate the response of epidermal growth factor (EGFR) after it binds to its cognate ligands. Additionally, we highlighted that the CD44v6/EGFR complex is correlated with lymph node metastasis and the human epidermal growth factor receptor 2 (HER2) breast cancer subtype and is inversely correlated with TN status [12]. CD44v3 was the second variant identified to interfere with growth factor signaling. CD44v3 effects are mainly due to its ability to bind heparin-binding growth factors such as macrophage inflammatory protein-1β [13], hepatocyte growth factor/scatter factor [14], fibroblast growth factor (FGF) [15], and heparin binding EGF like growth factor (HB-EGF) [16]. In a relevant study, we showed that NGF induces the complex-forming association of CD44 with the tyrosine kinase receptor TrkA, resulting in the activation of various signaling cascades that promote survival or migration/invasion [17]. However, to date, the identity of the CD44 variant that interacts with TrkA and precise mechanisms of this interaction and the intracellular action of this TrkA-binding CD44 variant are unknown. Therefore, in this study, we sought to determine whether CD44 variant 3 binds TrkA. We found that NGF induces direct binding between CD44v3 and TrkA. Additionally, we found that CD44v3 binds TrkA via the interaction of the CD44v3 C-terminal region and leucine-rich region 1 (LRR1) of TrkA. In a most unusual finding, we observed that CD44v3 cannot act as a coreceptor because it does not bind NGF. Specifically, using a CD44v3 C-terminal-mimicking peptide, we demonstrated that the interaction of CD44v3 with TrkA is critical for nerve growth factor (NGF)-induced phospho-TrkA-independent RhoA activation. Through a thorough investigation, we determined that the inhibition of the CD44v3-mimic/TrkA complex decreased tumor growth and metastasis *in vivo*. This outcome suggests that the CD44v3/TrkA interaction may be leveraged in breast cancer therapy because only cancer cells, not their normal counterparts, express this complex.

## Methods

### Cell culture

MDA-MB-231, SUM159-PT, T47-D, BT-474 and HCC-1954 breast cancer cell lines were purchased from the American Type Culture Collection (ATCC, Manassas, VA, USA). The COS-7 cell line (African green monkey kidney) was obtained from Pr. V. Orian-Rousseau (Karlsruhe Institute of Technology [KIT], Karlsruhe, Germany). MDA-MB-231 cells were cultured in Eagle’s minimal essential medium (EMEM), and COS-7 cells were cultured with Dulbecco’s minimal essential medium (DMEM) supplemented with 10% inactivated fetal bovine serum (FBS) (HyClone, Belgium), 1% nonessential amino acids, 40 UI/ml of penicillin/streptomycin and 40 μg/mL of gentamycin. SUM159-PT cells were cultured in F12 medium supplemented with 5% inactivated FBS, 1% HEPES, 100 UI/mL of penicillin/streptomycin, 40 μg/mL of insulin and 1 μg/mL of hydrocortisone. T47-D cells were cultured in RPMI+GlutaMAX supplemented with 10% inactivated FBS, 1% nonessential amino acids, 40 UI/mL of penicillin/streptomycin, 40 μg/mL of gentamycin and 10 μg/mL of insulin. BT-474 cells were cultured in RPMI supplemented with 40 UI/mL of penicillin/streptomycin and 10 μg/mL of insulin. HCC-1954 cells were cultured in RPMI supplemented with 10% inactivated FBS and 40 UI/mL of penicillin/streptomycin. All the cell lines were incubated at 37 °C in a humidified atmosphere containing 5% CO_2_. The cells were amplified after acquisition and cultured for no more than 25 passages. Before treatment, the cells were rinsed twice with trypsin/EDTA, cultured for 24 h in medium supplemented with 0.1% FBS, and then treated with recombinant human β-NGF (referred to as NGF and administered at 100 ng/ml in all experiments) (Alomone Labs, Israel) for the indicated times.

### Plasmids

To obtain plasmids containing the CD44 variant 3 or variant 6 sequence, the sequences were inserted into a pcDNA3.1/Hygro plasmid by polymerase chain reaction (PCR)-based mutagenesis. The plasmid carrying CD44v3 with a tagged histone (N-terminal) was produced by e-Zyvec. The pBabe-Puro-RhoA FLARE.sc biosensor was a gift from Klaus Hahn (Addgene plasmid # 12602; http://n2t.net/addgene:12602; RRID, Addgene_12,602) [18]. The cloning procedure is available upon request. Transient transfections were performed by electroporation using Amaxa® Nucleofector® technology (Lonza, Switzerland) according to the manufacturer’s instructions.

### In situ proximity ligation assay (PLA)

Cells (10,000 cells per well) were grown on acid-washed eight-well glass slides (Thermo Scientific) in the appropriate medium supplemented with 10% FBS for 24 h. After NGF treatment, paraformaldehyde (PAF)-fixed cells were incubated with 4% bovine serum albumin (BSA) or 5% FBS (for 1 h at 20 °C), followed by overnight incubation with primary antibodies (rabbit anti-TrkA or rabbit anti-IgG + mouse anti-pan-CD44; mouse anti-CD44v3; mouse anti-CD44v6; mouse anti-IgG2a; mouse anti-IgG2b; or mouse anti-IgG1). PLA was performed following the manufacturer’s instructions in the assay kit (Duolink PLA; Sigma–Aldrich). To obtain the correct representation of the PLA signal for each condition, 30 fields per condition were observed using a fluorescence microscope (Eclipse TiU; Nikon, Japan). The PLA signals (red dots) were automatically quantified using ImageJ software (with an in-house-developed plug-in). After PLA, statistical analyses were performed using GraphPad Prism software to determine the mean dot intensity in the cells.

Paraffin-embedded sections of breast tumors were obtained from US BioMax (HBre-Duc150Sur01). The sections were rinsed twice in Tris-buffered saline (TBS; 0.01 M Tris, pH 7.4; 0.15 M NaCl; and 0.05% Tween 20) and incubated with blocking solution (20% FBS in TBS for 1 h at room temperature [RT]). The slices were incubated with the same primary antibodies as those used for immunocytochemistry (cited above, diluted 1:50 in 1% FBS and incubated overnight at 4 °C).

### Protein extraction

Subconfluent cells were washed twice with ice-cold PBS and lysed (40 mM HEPES, pH 7.5; 120 mM NaCl; 1 mM EDTA; 1% Triton X-100; 0.1% SDS; 10% glycerol; 10 mM sodium pyrophosphate; 50 mM sodium fluoride; 1.5 mM sodium orthovanadate; and 1 mM PMSF, supplemented with a protease inhibitor cocktail) (Sigma–Aldrich, France). The cell lysates were cleared by centrifugation (6000 × g for 10 min at 4 °C) and stored at −80 °C until analysis. The supernatants were collected, and the protein concentration was determined using the bicinchoninic acid assay (Sigma–Aldrich).

### Microscale thermophoresis (MST)

MST was conducted using an NT.115 A Pico MST instrument (Nano Temper Technologies GmbH) equipped with red and blue filter sets. The TrkA-His tagged extracellular domain protein (Ala 34-Gly423) (9966-TK-050; R&D Systems, United Kingdom), diluted to 200 nM in PBS-T buffer (supplied by the vendor), was labeled using a Monolith His-Tag Labeling Kit with RED-Tris-NTA (Nano Temper). The RED-Tris-NTA dye was diluted in PBS-T to 100 nM. The mix was incubated at RT in the dark for 30 min. Next, an extract expressing CD44v3-labeled protein (CD44v3-His) and ligand and/or TrkA was mixed at a 1:1 ratio and incubated at RT in the dark for 15 min. For the binding check, peptides and TrkA-His were used. Capillaries were filled individually and loaded into the instrument. The data were acquired using high MST power and 100% LED. The data were analyzed using MO Control Software (Nano Temper).

### Production of the interaction model

Because the TrkA crystal structure is available, we directly retrieved it from the Protein Data Bank (PBD ID: 2IFG) at a resolution of 3.4 Å [19]. This structure was used to predict the interaction between TrkA and the peptide CD44v3, for which we generated many 3D structures. The interaction model was finally obtained using a protein–protein algorithm and, more specifically, the ClusPro2.0 webserver [20]. The subsequently obtained docking poses were minimized and scored.

### Selection of the residue target for mutagenesis

The residue target for mutagenesis was selected based on a visual inspection of the structure of the docking model with the best score and betweenness centrality analysis (BCA) was performed using a residual interaction network (RIN) generated from the PDB of the docking model form and native structure of TrkA. An RIN is defined as a network of interactions between the amino acids of a structure; thus, nodes are amino acids, and edges are the interactions detected between nodes. The RIN in this study was generated using an in-house C program, and residue-residue contacts were selected when the distance between them was between 2.5 Å and 5 Å. BCA was performed using the RINspector app [21, 22] of Cytoscape [23], and Z scores ≥2 were considered central residues. We focused on the residues at the interface of the protein that were deemed central to the interaction complex based on BCA but were not in the native TrkA structure, with the former criterion considered indicative of the essentiality of the residue for the interaction; only His112 fit this criterion. Therefore, His112 was selected for mutagenesis.

### Modeling of the peptide structures

For each peptide, the structure was produced *de novo* using the PEP-FOLD 3.5 webserver. The amino acid sequence of each peptide was used as input. Modeling was performed using standard parameters. The representation of the best model was then visualized and colored using Chimera software.

### Clonogenicity assay

The clonogenicity assay was performed to assess cell survival. MDA-MB-231 cells (250 cells/mL) were seeded into 6 plate wells (2% FBS medium; 2 mL/well). When the cells attached (within 3 h), they were treated with the peptide (scramble or P4; 50 ng/mL) or no peptide (control) and incubated for 24 h. The next day, a second-round treatment was performed. Three days later, the medium was changed, and the cells were incubated for another 5 days. After 10 days of culture, the medium was removed, and the cells were washed twice (PBS 1×), fixed (4% PAF for 30 min at RT), colored (violet crystal 2%; 30 min; RT) and washed twice (distilled water). Representative pictures of one well were taken, and the colonies were counted.

### Scratch/wound migration assay

Scratch/wound migration assays were performed using inserts (Ibidi, Germany). MDA-MB-231 cells (4 × 10^5^ cells/mL) were seeded onto each side of the chamber and incubated overnight at 37 °C until they reached the subconfluent stage. Next, the inserts were removed, and the cells were rinsed and starved (1 mL of 0.1% FBS medium for 1 h). Images of the initial wound were obtained using an inverted microscope, and the cells were incubated with or without the peptide (scramble or P4; 50 ng/mL) for 30 min and stimulated with NGF (100 ng/ml). After 24 h, images (2/wound) were obtained of the same place on the wound as those initially taken. The distance between the 2 migration fronts was measured using ImageJ software.

### Cell invasion assay

Transwell invasion assays were performed to assess cancer cell invasion in a Transwell chamber containing a collagen I-coated membrane (12-wells with inserts and 8 mm pore size membranes; Corning, Life Sciences). A total of 1 × 10^6^ starved cells (0.1% in SVF medium) were plated in the top chamber and incubated for 10 min before treatment with a peptide (50 ng/mL for 30 min); NGF was added to the lower chamber. After 24 h, the cells were fixed with methanol for 10 min and stained with Hoechst 33258 (1 mg/mL) for 30 min, and the cells that invaded the lower surface through the pores of the inserts were automatically counted under an inverted microscope with ImageJ software (with an in-house-developed plug-in).

### Förster resonance energy transfer (FRET)

MDA-MB-231 native or KD cells were seeded (5000 cells/well; ovn, 37 °C) in a chambered coverglass system (Lab-Tek®) coated with a type I collagen matrix (100 μg/mL; Corning). These cells were transfected using the pBabe-puro-RhoA plasmid (0.15 μg; Addgene plasmid #12602) diluted in a buffer solution (JetOPTIMUS® buffer) and a transfection agent (TA; 10 min). The mixture was incorporated into the culture medium (4 h; 37 °C). Two to three hours before image acquisition, the cells expressing the RhoA biosensor were starved in fresh EMEM containing 0.1% FBS and no phenol red. During image acquisition, the cells were maintained at 37 °C and with 5% CO_2_ in an incubation chamber installed under a microscope. Images were collected using a ZEISS LSM 880 confocal microscope with a plan-apochromat 63×/1.4 oil differential interference contrast (DIC) objective. The argon laser was tuned to emit 458 nm and 514 nm laser lines through a 470 to 500 nm bandpass emission filter (BP470–500) for CFP detection and a 530 nm longpass emission filter (LP530) for YFP detection. The emission of the CFP, FRET and YFP channels was recorded. The fluorescence emission ratios were computed using ImageJ software, which was also used to generate a report showing the FRET efficiency and intensity signal for each channel.

### Tumor xenograft growth in SCID (immunodeficient) mice

MDA-MB-231 cells (2 × 10^6^) were subcutaneously injected into the flanks of six-week-old female SCID mice. One month after injection, the mice were randomized into 2 groups and injected near the tumor with CD44v3 mimetic peptide 4 (P4) or not (control) five times in a 3-day interval. Tumor volume was determined throughout the experiment by measuring the length (l) and width (w) and was calculated as π/6 × l × w × (1 + w)/2. Animals were sacrificed using isoflurane anesthesia when the tumors reached a similar size, and the organs were immediately removed and stored in liquid nitrogen.

### RNA extraction from organs

Each organ was placed in a Precellys tube with QIAzol (1 mL) for lysis using a Precellys machine (3×). Next, RNA was extracted following the instructions of the Qiagen RNAeasy Mini Kit. All the procedures were performed on ice to prevent RNA degradation.

### Reverse transcription PCR

cDNA (5 ml) was mixed with Promega GoTaq kit components (GoTaq Buffer, dNTP, 10 μM primer, and GoTaqPol). After spinning down the contents, PCR cycles (95 °C for 2 min; 40 cycles of 95 °C for 30 s, 60 °C for 30 s, 72 °C for 30 s; 72 °C for 2 min; holding at 4 °C) were performed using a TC-5000 thermocycler. After the addition of the dye (6×), cDNA was separated in an agarose gel (2%).

### Statistical analyses

Statistical analyses were performed using GraphPad Prism 5.01 software. Analyses were performed using one-way analysis of variance (ANOVA) for the PLA, clonogenicity and Transwell assay data followed by Bonferroni’s posttest. For the wound assay, analyses were performed using two-way ANOVA with Bonferroni’s posttest. Tumor growth was analyzed by T test.

## Results

### CD44 variant 3 is involved in the TrkA/CD44 interaction following NGF stimulation

**Fig. 1:**
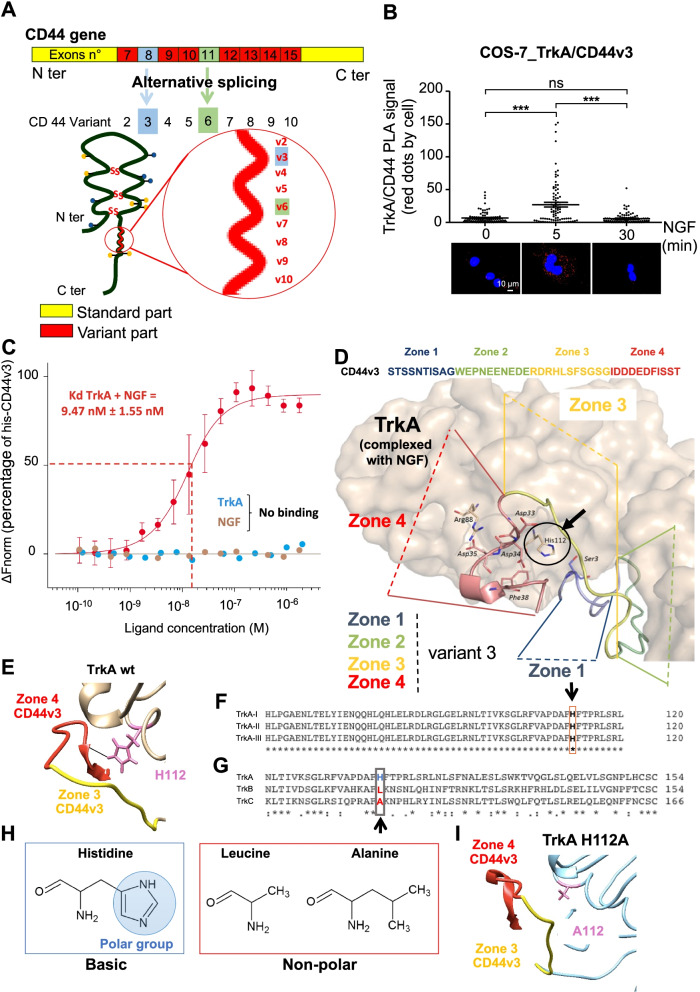
The C-terminal region of CD44 variant 3 binds to TrkA. **A** Schematic representation of the CD44 gene and protein structure. Alternative splicing leads to the expression of CD44 variants, thus the insertion of exon 8 leads to the expression of a CD44 protein containing variant 3 (CD44v3) and the insertion of exon 11 leads to the expression of a CD44 containing variant 6 (CD44v6) (**B**) TrkA/CD44v3 complex formation in COS-7 cells expressing TrkA and CD44v3 was detected by the proximity ligation assay (PLA), which was performed 0, 5 and 30 min after the initiation of NGF (100 ng/ml) treatment. The images represent signals detected by PLA. Quantification of the red spots was performed by ImageJ software (30 randomly chosen fields per condition of three different experiments). Statistical analyses were performed using one-way ANOVA followed by Bonferroni’s posttest. Error bars represent the standard error of the mean (S.E.M.); *** *p* < 0.001. ns, nonsignificant. **C** NGF and/or TrkA binding to CD44v3 was measured by microscale thermophoresis (MST). MST experiments were performed on whole cell extracts of COS-7 cells expressing CD44v3. **D**, **E** Modeling of TrkA and CD44 variant exon 3; sequence alignment of TrkA isoforms (**F**) or TrkA, TrkB and TrkC (**G**) using the Clustal Omega program. **H** Structure of histidine, leucine and arginine amino acids (ChemSketch software). **I** Modeling of TrkA H112A and CD44v3

In our previous studies, we demonstrated that NGF induces the association between TrkA and CD44 in cancer cells [17]. The CD44v3- and CD44v6-containing isoforms expressing respectively exon 8 and 11(Fig. 1A) can interact with growth factor receptors. Using COS-7 cells with low CD44 and TrkA expression (Supplementary Fig. 1A-D), we differentially expressed TrkA and CD44s or a CD44 variant, either CD44v3 or CD44v6 (Fig. 1B, supplementary Fig. 2). PLA revealed greater TrkA/CD44 variant associations within a complex (Fig. 1B). Notably, only the expression of CD44 variant 3 with TrkA increased the PLA-detected TrkA/CD44v3 signal in the plasma membrane of COS-7 cells following NGF stimulation. Additionally, formation of the TrkA/CD44v3 complex was transient and decreased 30 min after NGF stimulation (Fig. 1B). Neither wild-type COS-7 cells nor COS-7 cells transfected with TrkA and the CD44s or CD44v6 isoform showed the formation of the dynamic TrkA/CD44 complex in PLA, indicating that these two isoforms are not involved in complex formation (Supplementary Fig. 2A-C). Moreover, in MDA-MB-231 breast cancer cells, flow cytometric analysis also revealed that NGF increased the plasma membrane level of CD44v3 but not CD44v6 (Supplementary Fig. 3A-D). Additionally, we evaluated the expression of TrkA and CD44v3 at the membrane level or in cells by immunofluorescence in MDA-MB-231 cells. The level of CD44v3 increased at the membrane until 15 min of NGF treatment and then decreased (Supplementary Fig. 3E & F). Interestingly, the membrane level of TrkA followed the same trend. Additionally, the overall fluorescence intensities for TrkA and CD44v3 increased globally in the cell, suggesting that after internalization, the proteins were not degraded (Supplementary Fig. 3G & H).

The complex formation mechanism was then further examined. Because CD44v3 binds growth factors, we tested the ability of CD44v3 to bind NGF by microscale thermophoresis (MST). In contrast to FGF-1 (Supplementary Fig. 4), NGF did not bind to CD44v3. Additionally, TrkA binding to CD44v3 was not detected in the absence of NGF but was observed in the presence of NGF (Kd = 9.47 nM) (Fig. 1C). Taken together, these data suggested a direct interaction between TrkA in complex with NGF and CD44v3, but neither NGF nor TrkA alone could bind CD44v3.

**Fig. 2:**
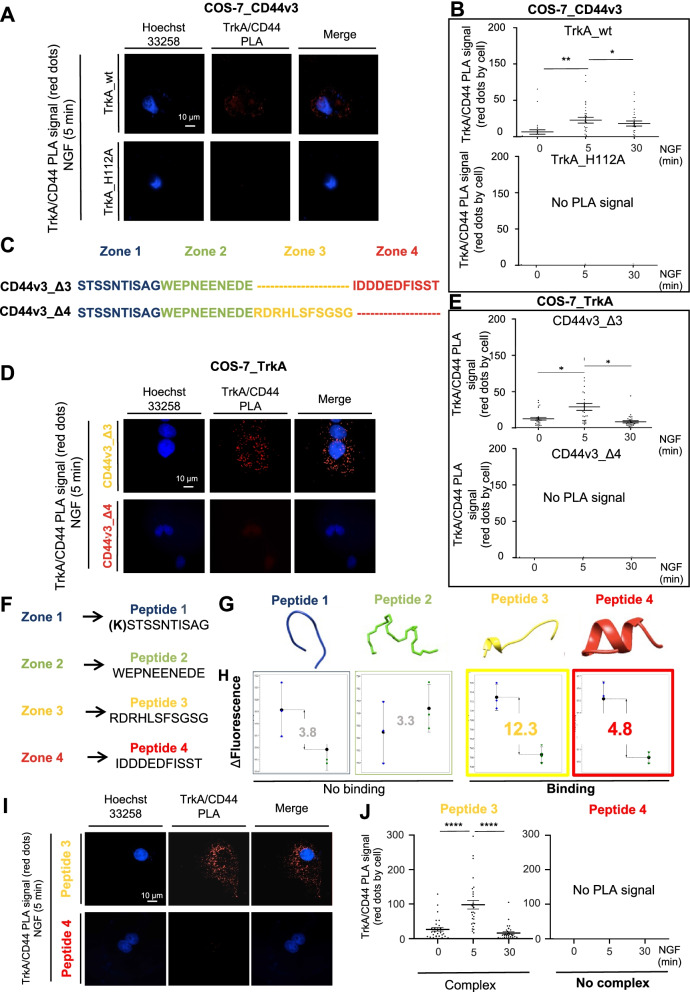
The C-terminal region of CD44v3 and the His112 residue of TrkA are involved in the CD44v3/TrkA interaction. TrkA/CD44v3 complex association was detected by the proximity ligation assay (PLA) in wild-type TrkA- or TrkA_H112A- and CD44v3-expressing COS-7 cells. Proximity ligation assays were performed after 0, 5 and 30 min of NGF treatment (100 ng/ml). Representative images of signals detected by PLA (**A**) and their quantification (**B**). Sequence of CD44 exon variant 3 and deletion mutant Δ3 or Δ4 (**C**). TrkA/CD44v3 complex association was detected by PLA in wild-type TrkA and CD44v3 wild type or CD44v3 deleted mutant Δ3 or Δ4 expressing COS-7 (**D**, **E**). Sequence of CD44v3 mimetic peptides (**F**) and structure (**G**). TrkA binding to CD44v3 mimetic peptides by microscale thermophoresis (**H**) and effects of CD44v3 mimetic peptides 3 (RDRHLSFSGSG) and 4 (IDDEDFISST) on TrkA/CD44v3 complex formation (**I**, **J**). Quantification of PLA was performed using ImageJ software (30 fields per condition of three different experiments). Statistical analyses were performed using one-way ANOVA followed by Bonferroni’s posttest. The error bars represent the standard error of the mean (S.E.M.); * *p* < 0.05; ** *p* < 0.01

To further investigate the interaction between TrkA and CD44v3, molecular modeling was performed using the available structure of TrkA complexed with NGF to identify amino acid residues involved in TrkA/CD44v3 complex formation (Fig. 1D). Because no structure of variant exon 3 was available, the CD44v3 sequence was cut into 4 domains of approximately 10 amino acid residues (zone 1, STSSNTISAG; zone 2, WEPNEENEDE; zone 3, RDRHLSFSGSG; and zone 4, IDDDEDFISST), and we scanned each peptide to locate the putative binding sequence. Visual examination of the TrkA-CD44v3 poses suggested that TrkA His112 binds to the C-terminal region of exon v3 (IDD^34^DEDF^38^ISST) (Fig. 1D & E). Statistical BCA confirmed that His112 in zone 4 of CD44v3 is likely a key residue for the interaction of this variant with TrkA (Supplementary Fig. 5A). This histidine residue was critical in all TrkA isoforms (I, II and III) (Fig. 1F) but not in TrkB or C (Fig. 1G). In the latter two receptors, His112 was replaced with either a leucine or alanine, and subsequent analysis indicated that His112 is highly specific to TrkA. Notably, His112 is a polar amino acid that can form a hydrogen bond with the C-terminal region of CD44v3 (Asp4 and Phe38) while leucine or alanine in TrkB and TrkC are non-polar (Fig. 1H). Interestingly, multiple sequence alignment with sequences of various mammals also showed that His112 is highly conserved among mammals (Supplementary Fig. 5B-D). Additionally, replacing this histidine with arginine in rabbits and mice, in which the TrkA sequence is similar, did not abolish the CD44v3-TrkA interaction. Therefore, the conservation of this histidine residue was investigated more extensively through phylogenic analysis (Supplementary Fig. 5E). TrkA is conserved across vertebrates; therefore, Trk sequences in species of each vertebrate taxon were aligned to its more distant ancestor, DTrk (*Drosophila melanogaster*). In vertebrates and *D. melanogaster*, histidine is replaced by 1 of 3 different polar residues (lysine, glutamine, or glycine), which can form a hydrogen bond (glutamine in *Danio rerio* and *Gallus*; lysine in *Branchiostoma floridae;* and glycine in *D. melanogaster*). These observations indicated the positive selection of histidine 112 and its physiological importance. Additionally, alignment analysis of the CD44v3 sequence with that of CD44v6 showed that a region of CD44v6 shared 42% identity with the C-terminal region of CD44v3. Interestingly, when the residues corresponding to Asp34 and Phe38 were replaced by glutamate, a weaker hydrogen bond was formed, and when they were replaced with proline, no hydrogen bond was formed, explaining the absence/weak association of TrkA/CD44v6 indicated by PLA (Supplementary Fig. 2C). These data indicated that TrkA H112 and the CD44v3 C-terminal region are likely crucial for the biological function of these respective proteins. Therefore, using COS-7 cells, we first tested the effect of the TrkA H112A mutation (Fig. 1I) on TrkA/CD44v3 complex formation (Fig. 2A & B). In the TrkA H112A mutant cells, TrkA binding to CD44v3 was lost, as indicated by the lack of a PLA signal in COS-7 cells, compared with TrkA wild-type cells, as detected by PLA, confirming the essential role of H112 in the TrkA-CD44v3 interaction (Fig. 2A & B). The involvement of the CD44v3 C-terminal region (IDDDEDFISST) in TrkA/CD44v3 complex formation was then tested. To complete this test, expression vectors of CD44v3 lacking its C-terminal region (IDDDEDFISST) or neighboring region (RDRHLSFSGSG), constructs named CD44v3_Δ3 and CD44v3_Δ4, respectively (Fig. 2C), were cotransfected into COS-7 cells with TrkA. The number of TrkA/CD44v3 complexes in these transfected COS-7 cells was then quantified by PLA (Fig. 2D & E). The CD44v3_Δ4 mutant could not bind TrkA because no signal was detectable by PLA, while CD44v3_Δ3 could bind TrkA following NGF stimulation to an extent similar to that of CD44v3 (Fig. 2D & E). Taken together, these results suggest that the C-terminal region of variant 3 is involved in TrkA binding and this is a general mechanism because it can be observed in breast cancer cells but also in cos7 in which the expression of TrkA and CD44 were induced.

### The C-terminal mimetic peptide of CD44v3 disrupts the TrkA-CD44v3 association

To block the TrkA-CD44v3 association, 4 mimetic peptides of CD44v3 were designed corresponding to zone 1 [(K) STSSNTISAG], zone 2 (WEPNEENEDE), zone 3 (RDRHLSFSGSG) and zone 4 (IDDDEDFISST) (Fig. 2F & G). The binding capacities of the CD44v3 mimetic peptides to TrkA were then measured by MST (Fig. 2H). As expected, neither peptide 1 (KSTSSNTISAG) nor peptide 2 (WEPNEENEDE) could bind TrkA, while mimetic peptide 4 (IDDDEDFISST) corresponding to the C-terminal region of CD44v3 could bind TrkA. To confirm mimetic peptide 4 binding, we assessed its ability to bind TrkA using nanoscale differential scanning fluorimetry (nanoDSF; Supplementary Fig. 6A & B). We observed that mimetic peptide 4 (but not a scramble peptide) bound TrkA, but we did not detect CD44v3/P4 binding (Supplementary Fig. 6C).

Unexpectedly, the RDRHLSFSGSG peptide (P3) corresponding to CD44v3 zone 3 also bound TrkA. Therefore, the abilities of peptides 3 and 4 to disrupt TrkA/CD44v3 complex formation were evaluated in the COS-7 cell line (Fig. 2I & J). As observed in CD44v3 mutants, only peptide 4 corresponding to zone 4 of CD44v3 could decrease NGF-induced TrkA/CD44v3 complex formation in COS-7 cells. However, peptide 3 did not inhibit TrkA/CD44v3 complex formation.

Collectively, our results demonstrated that the interaction of TrkA and CD44v3 was blocked by mimicking peptide 4 (IDDDEDFISST), which corresponds to the C-terminal region of exon variant 3 of CD44.

### The TrkA-CD44v3 interaction is involved in NGF-induced migration and invasion of MDA-MB-231 TNBC cells

**Fig. 3:**
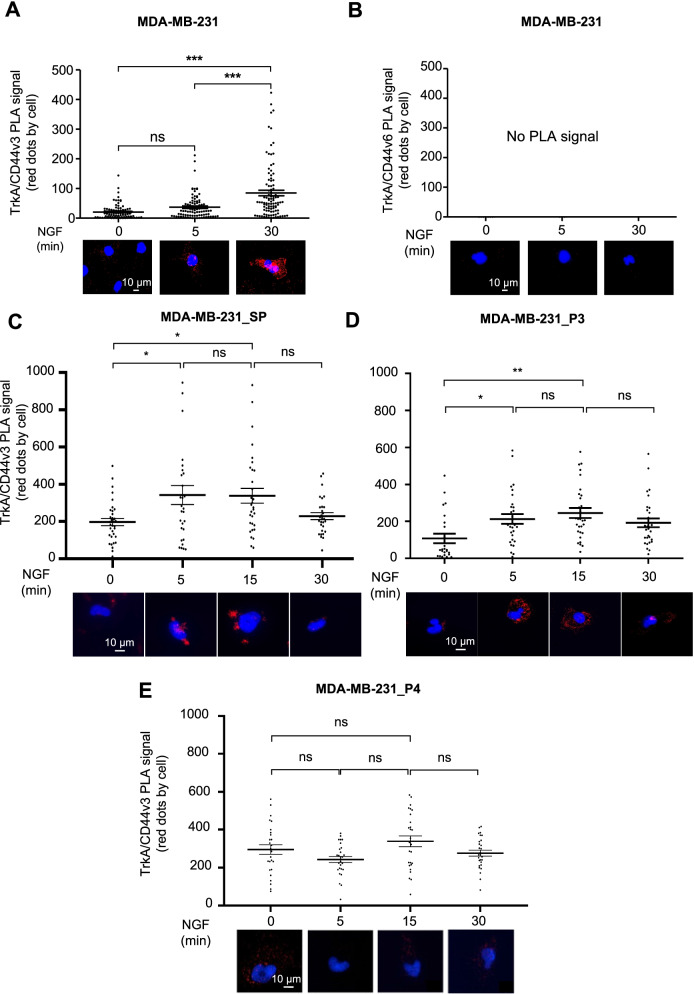
CD44v3 mimetic peptide 4 blocks the TrkA-CD44 association in MDA-MB-231 breast cancer cells. TrkA/CD44v3 (**A**) and TrkA/CD44v6 (**B**) complex associations were detected by the proximity ligation assay (PLA) in MDA-MB-231 breast cancer cells. Effects of a scramble peptide (SP) (**C**), CD44v3 mimetic peptide 3 (P3) and 4 (P4) (**D**) on TrkA/CD44v3 complex formation induced by NGF. Quantification of the PLA results was performed by ImageJ software (30 randomly chosen fields per condition of three different experiments). Statistical analyses were performed using one-way ANOVA followed by Bonferroni’s posttest. The error bars represent the standard error of the mean (S.E.M.); * *p* < 0.05, ** *p* < 0.01, *** *p* < 0.001; ns, not significant

**Fig. 4:**
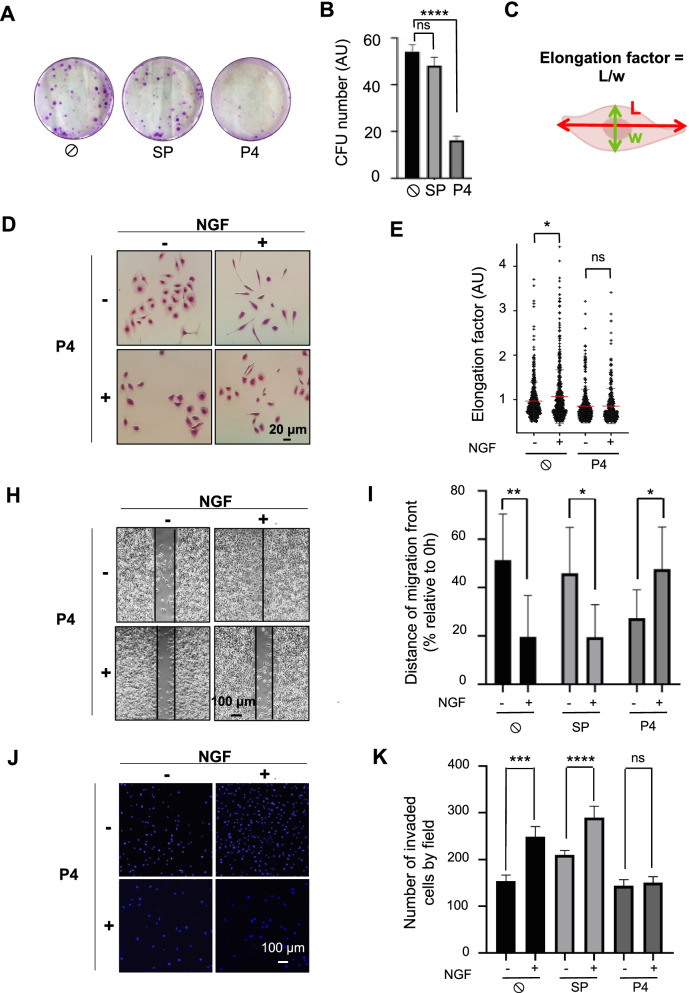
CD44v3 mimetic peptide 4 inhibits MDA-MB-231 breast cancer cell growth and migration/invasion. Clonogenic MDA-MB-231 cell growth was measured after 10 days in the presence of a scramble peptide (SP) or CD44v3 mimetic peptide (P4) (**A, B**). The elongation factor (length-to-width ratio) was assessed after 10 days in the presence or absence of NGF (**C**, **D**, **E**). Migration assays were performed using Ibidi devices 24 h after treatment (**H**, **I**). Invasion through collagen 1 was measured by the Transwell assay 16 h after treatment (**J**, **K**). Statistical analyses were performed using one-way ANOVA followed by Bonferroni’s posttest. The error bars represent the standard error of the mean (S.E.M.); * *p* < 0.05, ** *p* < 0.01, *** *p* < 0.001, **** *p* < 0.0001; ns, not significant

We previously showed that NGF is implicated in breast cancer aggressiveness because it enhances cell migration/invasion [17, 24]. Therefore, in this study, we assessed TrkA binding to CD44v3 in MDA-MB-231 cells, which were used as TNBC model cells. First, PLA was performed using anti-CD44 variant 3- (Fig. 3A and Supplementary Fig. 7) or anti-CD44 variant 6-specific antibodies (Fig. 3B). NGF induced TrkA/CD44v3 complex formation in MDA-MB-231 cells at the plasma membrane within 5 min to 30 min of administration (Fig. 3A and Supplementary Fig. 7) but did not induce TrkA/CD44v6 complex formation (Fig. 3B). Next, we tested the ability of CD44v3 mimetic peptides to block TrkA/CD44v3 complex formation in MDA-MB-231 cells (Fig. 3C-E). Similar to the results observed with COS-7 cells, neither a scramble peptide (Fig. 3C) nor mimetic peptide P3 (Fig. 3D) inhibited TrkA/CD44v3 complex formation following NGF treatment. Only mimetic peptide 4 (Fig. 3E) and the H112A mutation (Supplementary Fig. 8A & B) could disrupt TrkA/CD44v3 complex formation in MDA-MB-231 cells. Therefore, we evaluated the physiological consequences of TrkA/CD44v3 complex dissociation induced by CD44v3 mimetic peptide 4 on MDA-MB-231 cell phenotypes (Fig. 4). We first examined the effect of mimetic peptide 4 on clonogenic cell growth. For this experiment, MDA-MB-231 cells were grown in the presence of 2% FBS for 3 weeks in the presence or absence of CD44v3 mimetic peptide 4 (Fig. 4A and B). Under these conditions, CD44v3 mimetic peptide 4 reduced the proportion of MDA-MB-231 colony forming units by two-thirds compared with its effect on control cells or that of the scramble peptide. The same observations were made in SUM-159PT, another triple-negative cell line (Supplementary Fig. 9 A & B). Next, we tested the inhibitory effect of CD44v3 mimetic peptide 4 on the migration and invasion of MDA-MB-231 breast cancer cells (Fig. 4C-K). We first determined the effect of CD44v3 on the length-to-width (l/w) ratio of the cells (elongation factor) (Fig. 4C-E). NGF induced the acquisition of a fibroblast phenotype by MDA-MB-231 cells, which exhibited a significant increase in the l/w ratio (Fig. 4D-E). This effect of NGF was inhibited by the addition of CD44v3 mimetic peptide 4. Hence, MDA-MB-231 cell migration ability was assessed after NGF treatment in a wound healing test (Fig. 4H & I). Following NGF treatment, the migration of MDA-MB-231 cells was increased, as indicated by complete wound closure 24 h after treatment. This effect was not inhibited using the scramble CD44v3 mimetic peptide, while CD44v3 mimetic peptide 4 completely blocked NGF-dependent wound closure, indicating that CD44v3 mimetic peptide 4 inhibited the NGF-induced migration of MDA-MB-231 cells. We also confirmed the effects of CD44v3 mimetic peptide 4 on migration in SUM-159-PT cells (Supplementary Fig. 9 C & D). The MDA-MB-231 cell invasive capacity was then investigated using Boyden chambers (Fig. 4J & K). As observed in the pictures of the underside of the Transwell membrane (Fig. 4J), more NGF-treated cells than untreated cells invaded the collagen substrate. In the presence of CD44v3 mimetic peptide 4, a decrease in both basal levels and NGF-induced invasion was observed in MDA-MB-231 cells. These observations were confirmed by counting the cells that had invaded the Transwell membrane and were apparent on its underside (Fig. 4K). These effects on invasion were also observed in the MDA-MB-468 triple-negative breast cancer cell line (Supplementary Fig. 9 E). Additionally, the expression of TrkA-H112A also reduced migration and invasion compared with that of wild-type TrkA in MDA-MB-231 cells (Supplementary Fig. 8 C-E).

To determine the role of CD44v3 in NGF-induced phenotype acquisition, the effect of short interfering RNA (siRNA) targeting CD44v3 (siCD44v3) was assessed (Supplementary Fig. 10). siCD44v3 reduced the clonogenicity of MDA-MB-231 and SUM-159-PT cells (Supplementary Fig. 10 A & B) and MDA-MB-231 migration and invasion (Supplementary Fig. 10C-E).

Our results indicated that NGF also induced TrkA/CD44v3 complex formation in MDA-MB-231 breast cancer cells. Additionally, we depicted for the first time that the inhibition of TrkA/CD44v3 complex formation by CD44v3 mimetic peptide 4 is associated with the decreased clonogenic cell growth and migration/invasion abilities of MDA-MB-231 cells.

### TrkA/CD44v3 activates RhoA independently of TrkA phosphorylation

**Fig. 5:**
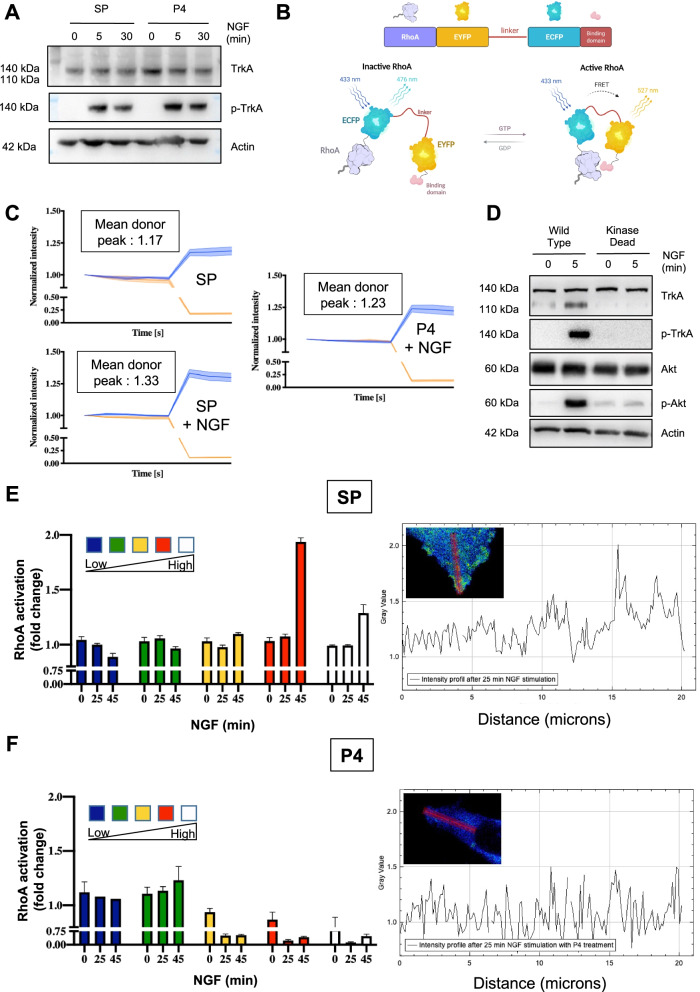
CD44v3 is implicated in the phospho-TrkA-independent activation of RhoA GTPase. Phosphorylation of TrkA was measured in the presence of a scramble peptide or CD44v3 mimetic peptide 4 (P4) in the presence or absence of NGF (**A**). Schematic representation of the Rho biosensor; (**B**). AB-FRET measurement of RhoA activation induced by NGF in MDA-MB-231 cells in the presence of a scramble peptide or P4 (**C**). Phosphorylation of TrkA and downstream Akt in wild-type MDA-MB-231 and MDA-MB-231 cells expressing kinase-dead TrkA, as determined by western blotting (**D**). Measurement of RhoA activation using the RhoA biosensor by FRET 25 and 45 min after NGF treatment in cells expressing either a scramble peptide (SP) or CD44v3 mimetic peptide 4 (P4). Based on the FRET signal intensity from low to high, the cells were divided into five groups (**E**, **F**). Localization of activated RhoA activation in MDA-MB-231 cells expressing kinase-dead TrkA (**G**, **H**)

To further elucidate the mechanism of action of CD44v3 mimetic peptide 4, we studied its effect on the TrkA signaling pathway. First, we performed western blot analysis to determine the effect of mimetic peptide 4 on TrkA phosphorylation (Fig. 5A). Interestingly, CD44v3 mimetic peptide 4 did not inhibit TrkA phosphorylation induced by NGF (Fig. 5A), indicating that disruption of TrkA/CD44v3 complex formation did not influence TrkA phosphorylation. Notably, we previously showed that NGF may enhance RhoA activity in breast cancer cells to promote migration/invasion independent of TrkA phosphorylation [17]. Therefore, we performed acceptor (photo) bleaching (AB)-FRET to examine the effect of CD44v3 mimetic peptide 4 on NGF-induced RhoA activity in MDA-MB-231 cells transfected with a RhoA FRET biosensor [18] (Fig. 5B). In MDA-MB-231 cells, NGF increased the FRET signal in the presence of the scramble peptide but not with CD44v3 mimetic peptide 4 (Fig. 5C). Additionally, to confirm that this effect was independent of TrkA phosphorylation, we measured RhoA activation in MDA-MB-231 cells expressing dead TrkA kinase, which cannot be phosphorylated (Fig. 5D). In these cells, we determined the ratiometric value of active (RhoA-GTP-to-yellow fluorescent protein (YFP) fluorescence) and inactive RhoA (RhoA-GTP-to-cyan fluorescence protein (CFP) fluorescence) after NGF stimulation (after 0, 25 and 45 min) and categorized the effect into five intensity categories (from low intensity, indicated in blue, to high intensity, indicated in white). We observed that NGF increased the proportion of active RhoA after 45 min in the presence of the scramble peptide (Fig. 5E) but not CD44v3 mimetic peptide 4 (Fig. 5F). Furthermore, we observed the relocation of activated RhoA to the migration front of the control cells with the scramble peptide after 25 min of NGF treatment but not with CD44v3 mimetic peptide 4 (Fig. 5E & F, Supplementary Fig. 11A). Furthermore, a similar decrease in RhoA activation was obtained using the TrkA-H112A mutation, which also prevents the formation of the TrkA/CD44v3 complex in the absence or presence of the TrkA kinase inhibitor k252A (Supplementary Fig. 11B & C, Supplementary Video 1 & 2).

Taken together, our results indicated that CD44v3 mimetic peptide 4 inhibits TrkA phosphorylation-independent RhoA activation induced by NGF.

### TrkA/CD44v3 is involved in tumor growth and MDA-MB-231 cell metastasis

**Fig. 6:**
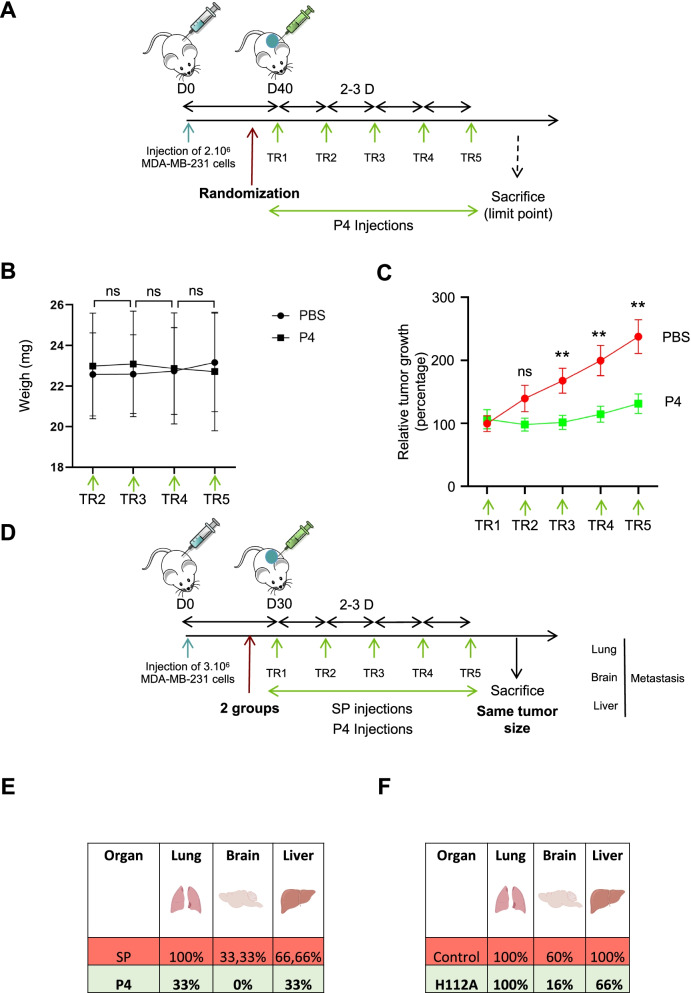
CD44v3 mimetic peptide 4 blocks MDA-MB-231 tumor growth and metastasis. Schematic representation of the experimental setup for MDA-MB-231 tumor growth assessment in SCID mice (**A**). Body weight (**B**) and tumor volume (**C**) were measured following CD44v3 mimetic peptide administration (10 mice per group). Schematic representation of the experimental setup for MDA-MB-231 metastasis assessment in SCID mice (**D**). Percentage of metastasis-positive lungs, brains and livers (**E**) after scramble peptide or CD44v3 mimetic peptide 4 treatment (3 mice/group) or (**F**) after TrkA-H112 mutant treatment (6 mice) compared with HA-TrkA cells (5 mice). ** *p* < 0.01; ns, nonsignificant. T test

To assess the role of TrkA/CD44v3 *in vivo*, SCID mice were injected with MDA-MB-231 cells to generate xenografts. Primary tumor development and metastasis (to the lung, brain and liver) were analyzed (Fig. 6). To determine the effects of TrkA/CD44v3, tumor cell growth was compared after treatment with mimetic peptide 4 and PBS (Fig. 6A-C). We first observed that CD44v3 mimetic peptide 4 did not affect the overall body weight of the mice (Fig. 6B). The development of primary tumors was measured throughout the experiments. CD44v3 peptide 4 injections were performed when the mean tumor volume reached 100 mm^3^ (Fig. 6C). The CD44v3 mimetic peptide significantly inhibited the growth of MDA-MB-231 cells in xenograft tumors. This result confirmed our preliminary clonogenic assay results, indicating that CD44v3 mimetic peptide 4 decreased the long-term growth of MDA-MB-231 cells both in vitro and *in vivo*. We also assessed the metastatic burden. Because CD44 mimetic peptide 4 blocked primary development, we chose to inject the CD44v3 mimetic peptide into the mice harboring the largest tumors. Hence, the mice were sacrificed when the primary tumors reached the same size (Fig. 6D). Micrometastases were detected at the end of the experiment by PCR of human microglobulin in xenograft mice treated with or without the CD44v3 mimetic peptide (Fig. 6E). Similar to previously reported results [17], our experiments showed that wild-type MDA-MB-231 cells colonized the lung and liver and, to a lesser extent, the brain. Interestingly, CD44v3 mimetic peptide 4 decreased the metastatic burden at each analyzed location. These results indicated that MDA-MB-231 cell metastasis in these three organs was blocked by CD44v3 mimetic peptide 4. To confirm the implication of the TrkA/CD44v3 complex in metastasis, we also performed xenograft experiments using MDA-MB-231 HA-TrkA H112A cells (Fig. 6F). We confirmed that HA-TrkA cells (control) appeared more metastatic than MDA-MB-231 cells [24], but MDA-MB-231 cells overexpressing TrkA H112A xenografted mice seemed to exhibit less metastasis in the brain and liver. These results suggested that the TrkA/CD44v3 association could be implicated in metastasis and that CD44v3 mimetic peptide 4 might impair metastasis development.

### The TrkA/CD44v3 complex is differentially detected in breast cancer cells and tumors

**Fig. 7:**
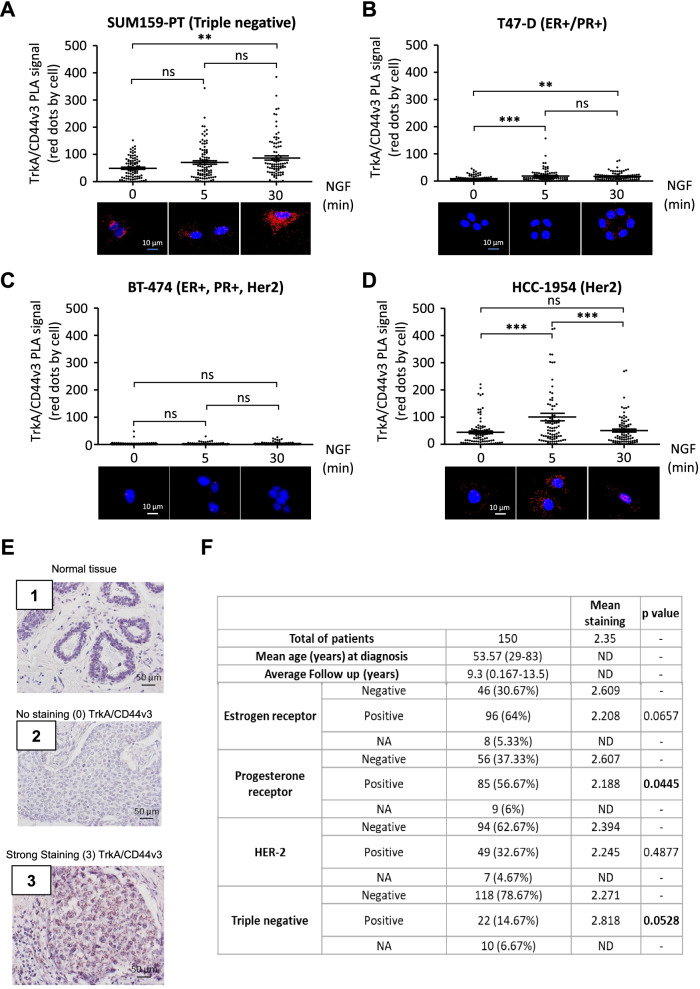
The TrkA/CD44v3 complex is formed in PR-negative breast cancer cells and tumors. TrkA/CD44v3 complex formation was measured in SUM159-PT (triple negative—i.e., ERα-, PR-, and Her2-) cells (**A**), T47-D (luminal A—i.e., ERα+, PR+, and Her2-) cells (**B**), BT-474 (luminal B—ERα+, PR+, and Her2+) cells (**C**), HCC-1954 (Her2-like—ERα-, PR-, and Her2+) cells (**D**), and 150 tumor patient samples obtained from US BioMax (HBre-Duc150Sur01) (**E**, **F**) using the proximity ligation assay (PLA). For breast cancer cell lines, quantification of the PLA results was performed using ImageJ software (30 randomly chosen fields per condition of three different experiments). Statistical analysis was performed using one-way ANOVA followed by Bonferroni’s posttest. The error bars represent the standard error of the mean (S.E.M.); * *p* < 0.05; ** *p* < 0.01; ns, not significant. Representative micrograph of PLA staining of normal tissue (**E**, **1**) and breast tumors (**E**, **2** and **3**). For PLA, the signals were classified into 4 categories ranging from no signal (**E**,**2**) to a high intensity signal (**E**,**3**). The scoring for TrkA/CD44v3 PLA staining is reported in Table (F) according to estrogen receptor, progesterone receptor, Her2 expression and triple-negative status (absence of estrogen receptor, progesterone receptor and Her2 overexpression). NA, information not available

NGF induced TrkA/CD44v3 complex formation in MDA-MB-231 cells, which are the gold standard models of TNBC. We also examined the expression and induction of TrkA/CD44v3 in breast cancer cell lines corresponding to different breast cancer subtypes (Fig. 7A-D). Four cell lines were assessed: SUM159-PT (TN; i.e., ERα-, PR-, and Her2-) cells, T47-D (luminal A; i.e.*,* ERα+, PR+, and Her2-) cells, BT-474 (luminal B; ERα+, PR+, and Her2+) cells and HCC-1954 (Her2-like; ERα-, PR-, and Her2+) cells. Under these conditions, NGF induction of CD44v3/TrkA complex formation was observed in SUM-159PT (TN) and HCC-1954 (Her2) cells. By contrast, T47-D cells (luminal A) expressed the TrkA/CD44v3 complex at a lower rate, and NGF induction was insufficient to modulate TrkA/CD44v3 complex formation in BT-474 (luminal B) cells, which are known to express a very low level of complex (compared with background noise in PLA). Therefore, TrkA/CD44v3 complex formation was likely induced differently among breast cancer cell lines. The TrkA/CD44v3 complex formation rate and activity level appeared higher in breast cancer cell lines, representing various breast cancer subtypes that did not express the progesterone receptor (PR). Therefore, TrkA/CD44v3 complex formation was assessed in breast cancer tissue microarrays to determine the level to which it correlated with clinical-biological parameters of breast tumors (i.e., estrogen receptor, PR and HER2 expression) (Fig. 7E & F). Complex formation was detected by PLA and scored ranging from no staining (0) (Fig. 7, E2) to high intensity staining (3) (Fig. 7, E3). Interestingly, we observed that the TrkA/CD44v3 complex was absent in “normal adjacent tissue” (Fig. 7, E1), indicating that TrkA/CD44v3 complex staining was specific to cancer cells. Using our scoring method, PR-negative tumors exhibited significantly higher TrkA/CD44v3 complex levels (mean staining: 2.607) than progesterone-positive tumors (mean staining: 2.188) (Fig. 7F). These results indicated that TrkA/CD44v3 complex formation is inversely correlated with PR. TrkA/CD44v3 staining in PLA was more intense in estrogen receptor-negative cells than in estrogen receptor-positive cells and in triple-negative cells than in non-TN cells; these correlations were at the limit of significance (p = 0.0657 and 0.0528, respectively).

## Discussion

**Fig. 8:**
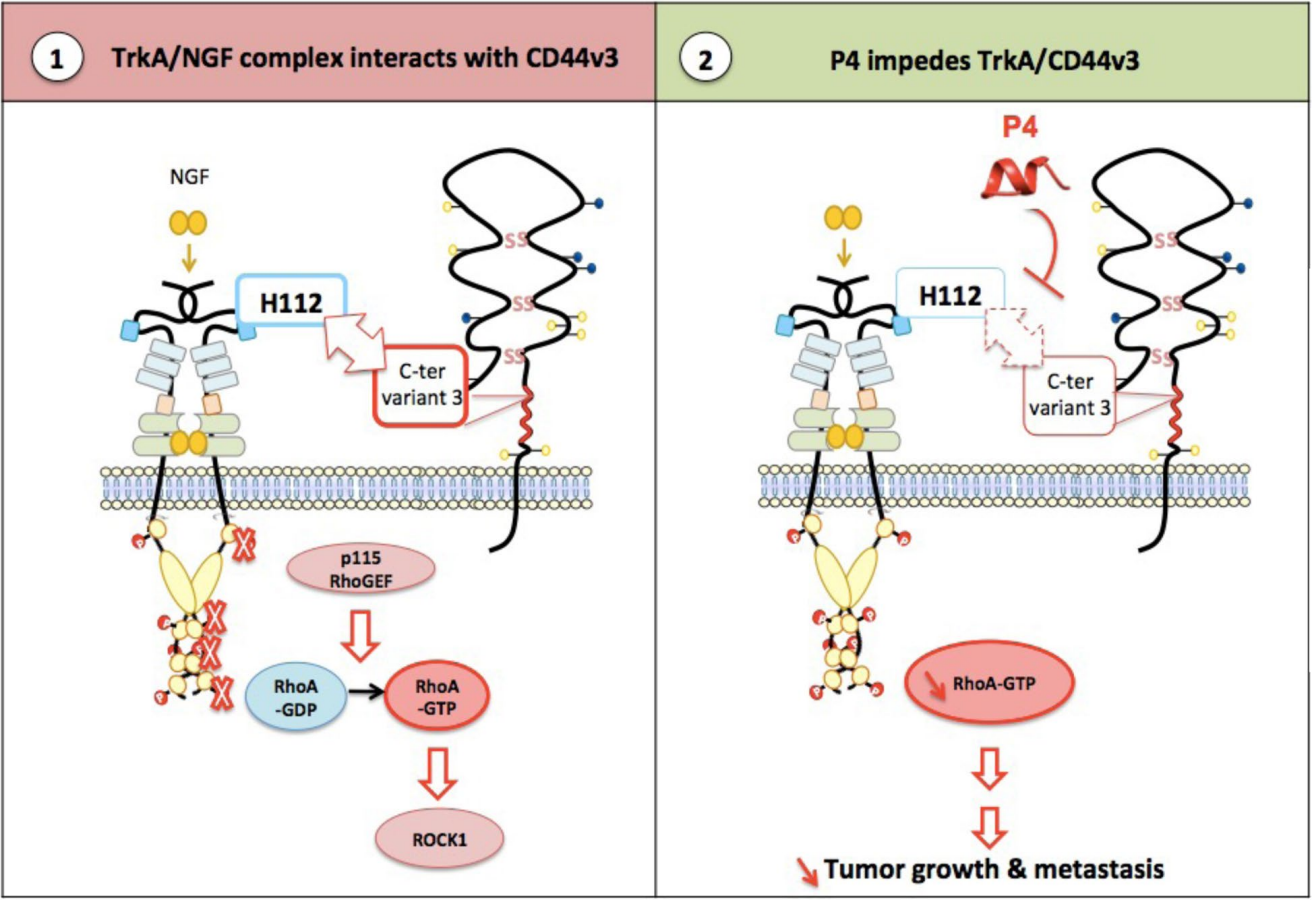
Schematic representation of the TrkA-CD44v3 interaction induced by NGF. (1) In the presence of NGF, TrkA interacts with CD44v3 variant 3 at histone residue H112. This interaction is the basis for phospho-independent TrkA signaling. It involves the p115 RhoGEF/RhoA/ROCK1 signaling cascade to induce tumor development. (2) In the presence of CD44v3 4 mimetic peptide (P4), the TrkA/CD44v3 complex cannot form, and phosphorescent-independent TrkA signaling through RhoA is inhibited. This results in decreased tumor development and metastatic capabilities

With more functions than those of a membrane receptor, CD44 is a multifunctional platform implicated in numerous physiological and disease mechanisms. In cancer, it has many functions (e.g., adhesion, migration/invasion, growth, differentiation, and resistance to therapies) depending on the splice variant expressed, corresponding ligands, posttranscriptional modifications, and association with growth factors and different membrane proteins. In the present study, we demonstrated, for the first time, the role played by CD44v3 in association with TrkA in the survival, migration, invasion, tumor growth and metastasis of breast cancer cells. After in-depth analysis, we described CD44v3 binding to TrkA as an original mechanism. The protein core of CD44v3 may bind directly to TrkA, and this binding does not involve a heparan sulfate moiety (Fig. 8). Hence, 10 amino acids in the C-terminal region of CD44 variant 3 likely engage the His112 residue in the LRR1 of TrkA. To our best knowledge, no description of the interaction between TrkA and/or its ligands with CD44v3 or other CD44 variants has been described. As recently shown, homophilic interactions of the CD44 protein in adjacent cancer cells are involved in the aggregation and metastasis of breast cancer cells. Amino acid residues from position 21 to 97 in the nonvariable region of CD44 were identified as critical for CD44 interactions [25]. The unique direct interaction of the protein core of CD44 with a growth factor receptor has been reported by Bourguignon *et al.* (1997) [26]. They reported that Her2 may be linked to the standard form of CD44 because of interchain disulfide bridge formation between the two receptors. More commonly, CD44v3 acts as a coreceptor that presents ligands to the conjugate receptor. This presentation requires the heparan sulfate chain of CD44v3, which binds heparin-binding growth factors [14]. For example, Yu *et al.* (2002) showed that the heparan sulfate chain of CD44v3 is involved in the formation of Hb-EGF, MMP7 and the ErbB4 complex at the cell surface [16]. However, NGF likely did not bind directly with CD44v3. This result aligns with the finding that, in contrast to other growth factors, NGF does not bind heparin [27]. Furthermore, the heparan sulfate-binding branch sequence is SGSG [28], which is located in CD44v3 region 3 (RDRHLSFSGSG), and we showed that the deletion of this region (CD44v3_Δ3) does not impede the NGF-induced association between CD44v3 and TrkA. Additionally, indirect effects were assessed by MST. A zone 3 mimetic peptide of CD44v3 (P3), which was not glycosylated, bound TrkA but did not abolish complex formation. Taken together, these findings suggest that CD44v3 glycosylation may not be involved in the TrkA-CD44v3 interaction.

CD44 is the main hyaluronan receptor, and it is permissive of interactions between cells and the extracellular matrix. However, CD44v3 recruitment of membrane proteins and receptors triggers multiple actions. As we highlighted in our previous study, CD44v3 may act as a signaling scaffold protein for TrkA. Specifically, we showed that NGF-induced CD44v3 binding to TrkA is necessary to activate RhoA GTPase at the leading edge of the cells and may be involved in directing migration [29].

As previously reported, TrkA overexpression enhanced the metastasis of MDA-MB-231 cells [24, 30]. This effect is attributed to the phosphorylation of TrkA, as NGF induced TrkA phosphorylation and subsequent signaling potentially involved in migration/invasion, and TrkA overexpression induced ligand-independent phosphorylation. Interestingly, the results presented herein seemed to invalidate these previous findings. In the present study, we showed that 1) CD44v3 mimetic peptide 4, which did not inhibit TrkA phosphorylation but blocked TrkA/CD44v3 complex formation, impeded tumor growth and metastases and that 2) RhoA activation was probably dependent not only on TrkA phosphorylation but also on complex formation by recruiting RhoA, as indicated by experiments in which we used kinase-dead TrkA (Fig. 8). These observations are interesting because they confirm that TrkA may be critical for breast cancer cell metastasis, not only through phosphorylation but also likely through its ability to heterodimerize with CD44v3.

Furthermore, our tissue microarray revealed that TrkA/CD44v3 is expressed only in cancer cells and not in normal epithelial cells. This finding is consistent with normal breast epithelial cells not expressing CD44v3 [31] but low levels of TrkA [24]. This first observation indicates that we could consider a treatment that targets the complex (similar to peptide 4) because it is specific to cancer cells and not normal cells. Furthermore, we determined that TrkA/CD44v3 complex formation is higher in PR-negative than PR-positive tumors, and a strong tendency (*p* = 0.0528) exists for the presence of the complex to correlate with triple-negative status in breast cancer and inversely correlate with the estrogen receptor (*p* = 0.0657). Thus, in Her2-positive cells, a subpopulation of PR-positive (luminal B) cells exists and the expression of Her2-positive cells does not allow the correlation of TrkA/CD44v3 formation with TN status. This inverse correlation between the presence of the TrkA/CD44v3 complex and progesterone receptor is consistent with the inverse correlation of TrkA expression with PR expression [17]. Additionally, CD44v3-containing isoforms are mainly associated with tumorigenesis [32] and breast cancer aggressiveness [33, 34]. Batsché *et al*. [35] also dissected the mechanism of CD44 variant inclusion and splicing and showed that variant inclusion is increased during tumor progression and metastasis in breast cancer.

In conclusion, we revealed a newly discovered mechanism of action of CD44v3 that is independent of its heparan sulfate chain. Additionally, the direct interaction between CD44v3 and TrkA leads to TrkA signaling that is independent of tyrosine phosphorylation. More specifically, the CD44v3-TrkA interaction is critical for breast cancer cell tumor development and metastasis *in vivo,* as indicated by the results showing that inhibition of TrkA/CD44v3 formation was sufficient to decrease the tumor burden and metastasis in SCID mice. Additionally, using CD44v3 mimetic peptide 4, therapeutic intervention is possible and may be a novel and interesting way to target TrkA oncogenic effects in tumors. Furthermore, developing bispecific antibodies targeting the complex or the treatment combination of TrkA and CD44v3 inhibitors is warranted. In breast cancer, TrkA/CD44v3 complex formation is inversely correlated with PR expression and is associated with TN status. In this latter subtype, only sacituzumab govitecam passed through drug process development and was recently approved by the Food and Drug Administration and European Medecines Agency [36]. Similar to sacituzumab govitecam, our fundamental discovery may highly impact the development of new treatments based on TNBC-specific membrane receptor targeting.

## Conclusion

Our results are highly original because CD44v3 binds to TrkA independently of its heparan sulfate and diversifies TrkA signaling through a TrkA phosphorylation-independent signaling pathway. Together, these results demonstrate that the oncogenic action of TrkA is linked to its association with CD44v3. Therefore, our therapeutic strategies to inhibit these receptor platforms must be reviewed.

## Supplementary Information

**Additional file 1: Supplementary video 1.** Measurement of RhoA activation using the RhoA biosensor by Förster resonance energy transfer (FRET) in the presence of a scramble peptide (SP) after NGF treatment of MDA-MB-231 cells expressing kinase-dead TrkA.

**Additional file 2: Supplementary video 2.** Measurement of RhoA activation using the RhoA biosensor by Förster resonance energy transfer (FRET) in the presence of CD44v3 mimetic peptide 4 (P4) after NGF treatment of MDA-MB-231 cells expressing kinase-dead TrkA.

**Additional file 3: Supplementary Figure 1.** Validation of the ectopic expression of CD44 and/or TrkA in COS-7 cells. The expression of all CD44 isoforms **(A)**, CD44 variant 3 **(B)**, CD44 variant 6 **(C)** and TrkA (hyaluronan [HA]; **D)** was evaluated by RT–qPCR (normalized using PUM-1). No expression of CD44, CD44 variants [3 or 6] or TrkA was detected in COS-7 cells **(A-D)** compared with that in cells transfected with the expression plasmid carrying each protein. **Supplementary figure 2.** TrkA does not interact with CD44s or CD44v6. Wild-type cos7 cells **(A)** or CD44S- **(B)** or CD44v6 **(C)** cells transfected with TrkA were treated with 100 ng/ml NGF (0, 5 or 30 min) and fixed. Quantification of the PLA results was performed using ImageJ software (30 randomly chosen fields per condition of three different experiments). Statistical analyses were performed using one-way ANOVA followed by Bonferroni’s posttest. The error bars represent the standard error of the mean (S.E.M.); ns, not significant. **Supplementary Figure 3.** CD44v3 and TrkA are recruited to the plasma membrane through NGF stimulation. CD44v3 and CD44v6 levels at the plasma membrane were assessed by flow cytometry **(A-D)**. The plasma membrane and total TrkA and CD44v3 levels were also quantified by confocal microscopy **(E-H).** Statistical analyses were performed using one-way ANOVA followed by Bonferroni’s posttest. The error bars represent the standard error of the mean (S.E.M.); * *p* < 0.05; ns, not significant. **Supplementary Figure 4.** NGF does not bind to CD44v3. The CD44v3/NGF interaction was evaluated using microscale thermophoresis (MST). FGF-2 (control) binding to CD44v3 but not to NGF was detected. **Supplementary Figure 5.** His112 of TrkA is conserved across mammals. Graphical representation of the interactions between residue H112 of TrkA and CD44v3 using betweenness centrality analysis (BCA) **(A)**. Conservation of His112 across mammals was assessed by alignment analysis of TrkA sequences in different mammals using WebLogos **(B)** and the Clustal Omega program **(C).** A phylogenetic tree using the neighbor-joining method constructed with the MegaX program shows the evolution of the TrkA His112 region across vertebrates. For each species, the residue corresponding to His112 was reported **(D)**. Schematic representation (ChemSketch) of the amino acid substitution of His112 across vertebrates **(E). Supplementary Figure 6.** CD44v3 mimic peptide 4 (P4) binds to TrkA but not to CD44v3. Peptide binding (P4; **A**) or not (scramble protein [SP]; **B**) was validated by nanoscale differential scanning fluorimetry (NanoDSF). The CD44v3/P4 with TrkA interaction was evaluated using microscale thermophoresis (MST; binding determination analysis is shown in **C**. **Supplementary Figure 7.** Plasma membrane TrkA/CD44v3 complex formation is transient. TrkA/CD44v3 complex association was detected using the proximity ligation assay (PLA) in MDA-MB-231 breast cancer cells. Quantification of the PLA results was performed using ImageJ software (30 randomly chosen fields per condition of three different experiments). Statistical analyses were performed using one-way ANOVA followed by Bonferroni’s posttest. The error bars represent the standard error of the mean (S.E.M.); *** *p*<0.0001; ns, not significant. **Supplementary Figure 8.** Effects of H112A mutation on the TrkA/CD44v3 complex. TrkA/CD44v3 complex association was quantified by the proximity ligation assay (PLA) in wild-type TrkA- or TrkA_H112A- MDA-MB-231 cells. Proximity ligation assays were performed after 0, 5 and 30 min of NGF treatment (100 ng/ml) **(A)**. Migration assays were performed using Ibidi devices 24 h after treatment **(B**, **C**). Invasion through collagen 1 was measured using the Transwell assay 16 h after treatment (**D**, **E**). Statistical analyses were performed using one-way ANOVA followed by Bonferroni’s posttest. The error bars represent the standard error of the mean (S.E.M.); * *p* < 0.05, **** *p*<0.0001; ns, not significant. **Supplementary Figure 9.** Effects of CD44v3 mimetic peptide 4 on triple-negative breast cancer cell growth and migration/invasion. Clonogenic SUM-159-PT cell growth was measured after 10 days in the presence of a scramble peptide (SP) or CD44v3 mimetic peptide (P4) **(A**, **B)**. Migration assays were performed using Ibidi devices 24 h after treatment (**C**, **D**). Invasion through collagen 1 of MDA-MB-468 cells was measured using the Transwell assay 16 h after treatment (**E**). Statistical analyses were performed using one-way ANOVA followed by Bonferroni’s posttest. The error bars represent the standard error of the mean (S.E.M.); * *p* < 0.05, ** *p*< 0.01, *** *p*<0.001; ns, not significant. **Supplementary Figure 10.** siCD44v3 inhibits Triple negative breast cancer cell growth and migration/invasion. MDA-MB-231 cells transfected (24 h) with short interfering RNA against CD44v3 (siCD44v3) or a control (siCTL) were used to perform clonogenic cell growth, migration and invasion assays. Clonogenic cell growth (MDA-MB-231)**(A)** and SUM-159-PT **(B)** was measured 10 days after siRNA transfection. Migration assays were performed using Ibidi devices 24 h after treatment **(C, D)**. The extent of cell invasion through collagen 1 was measured using the Transwell assay 16 h after treatment **(E)**. Statistical analyses were performed using one-way ANOVA followed by Bonferroni’s posttest. The error bars represent the standard error of the mean (S.E.M.); * *p* < 0.05, *** *p*<0.001, **** *p*<0.0001; ns, not significant. **Supplementary Figure 11.** CD44v3 mimic peptide 4 (P4) impedes local RhoA activation independent of TrkA phosphorylation. A) Measurement of RhoA activation using the RhoA biosensor by Förster resonance energy transfer (FRET) in the presence of a scramble peptide (SP) (supplementary video 1) or CD44v3 mimetic peptide 4 (P4) (supplementary video 2) after NGF treatment of MDA-MB-231 cells expressing kinase-dead TrkA. **(B**, **C)** Measurement of RhoA activation using the RhoA biosensor by FRET 25 and 45 min after NGF treatment in cells expressing either HA-TrkA or HA-TrkAH112A in the absence **(B)** or presence **(C)** of k252A (TrkA kinase inhibitor). Based on the FRET signal intensity from low to high, the cells were divided into five groups.

## Data Availability

The data and materials generated in this study are available upon reasonable request from the corresponding author: Prof. Robert-Alain Toillon, Université de Lille, Faculté des Sciences et Technologies, UMR CNRS 9020- INSERM U1277 - CANTHER - Cancer Heterogeneity Plasticity and Resistance to Therapies, Bâtiment SN3, 3ème étage, Cité scientifique, 59655 Villeneuve d’Ascq, France. Phone: +33 (0)3 20 43 65 59. E-mail: robert-alain.toillon@univ-lille.fr

## Abbreviations

BCA: Betweenness Centrality Analysis
BSA: Bovine Serum Albumin
CFP: Cyan Fluorescent Protein
CD44v: CD44 variant
DMEM: Dulbecco Minimum Essential Medium
EGFR: Epidermal Growth factor
EMEM: Earle’s salt Minimum Essential Medium
FBS: Fetal Bovine Serum
FGF: Fibroblast Growth Factor
FRET: Fluorescence Resonance Energy Transfer
HB-EGF: Heparin Binding EGF Like Growth Factor
HER2: Human Epidermal growth factor Receptor 2
HGF/SF: Hepatocyte Growth Factor/Scatter factor
LRR1: Leucin Rich Region 1
MIP1b: Macrophage inflammatory protein-1β
MST: MicroScale Thermophoresis
NGF: Nerve Growth Factor
PBS-T: Phosphate-buffered saline – Tween 20
PLA: Proximity Ligation Assay
RPMI: Roswell Park Memorial Institute
SCID: Severe Combined ImmunoDeficiency
SD: Standard Deviation
TBS: Tris-buffered saline
TN: Triple Negative
TNBC: Triple Negative Breast Cancer
TrkA: Tropomyosin kinase A
YFP: Yellow Fluorescent Protein
