# Supplementary material for: Direct interaction of TrkA/CD44v3 is essential for NGF-promoted aggressiveness of breast cancer cells
Source: J Exp Clin Cancer Res. 2022 Mar 28;41:110. doi: 10.1186/s13046-022-02314-4 (PMC8962522; doi:10.1186/s13046-022-02314-4)
Supplement: Supplementary file 3 — Additional file 3: Supplementary Figure 1. Validation of the ectopic expression of CD44 and/or TrkA in COS-7 cells. The expression of all CD44 isoforms (A), CD44 variant 3 (B), CD44 variant 6 (C) and TrkA (hyaluronan [HA]; D) was evaluated by RT–qPCR (normalized using PUM-1). No expression of CD44, CD44 variants [3 or 6] or TrkA was detected in COS-7 cells (A-D) compared with that in cells transfected with the expression plasmid carrying each protein. Supplementary figure 2. TrkA does not interact with CD44s or CD44v6. Wild-type cos7 cells (A) or CD44S- (B) or CD44v6 (C) cells transfected with TrkA were treated with 100 ng/ml NGF (0, 5 or 30 min) and fixed. Quantification of the PLA results was performed using ImageJ software (30 randomly chosen fields per condition of three different experiments). Statistical analyses were performed using one-way ANOVA followed by Bonferroni’s posttest. The error bars represent the standard error of the mean (S.E.M.); ns, not significant. Supplementary Figure 3. CD44v3 and TrkA are recruited to the plasma membrane through NGF stimulation. CD44v3 and CD44v6 levels at the plasma membrane were assessed by flow cytometry (A-D). The plasma membrane and total TrkA and CD44v3 levels were also quantified by confocal microscopy (E-H). Statistical analyses were performed using one-way ANOVA followed by Bonferroni’s posttest. The error bars represent the standard error of the mean (S.E.M.); * p < 0.05; ns, not significant. Supplementary Figure 4. NGF does not bind to CD44v3. The CD44v3/NGF interaction was evaluated using microscale thermophoresis (MST). FGF-2 (control) binding to CD44v3 but not to NGF was detected. Supplementary Figure 5. His112 of TrkA is conserved across mammals. Graphical representation of the interactions between residue H112 of TrkA and CD44v3 using betweenness centrality analysis (BCA) (A). Conservation of His112 across mammals was assessed by alignment analysis of TrkA sequences in different mammals using WebLogos (B) [file 13046_2022_2314_MOESM3_ESM.pdf]

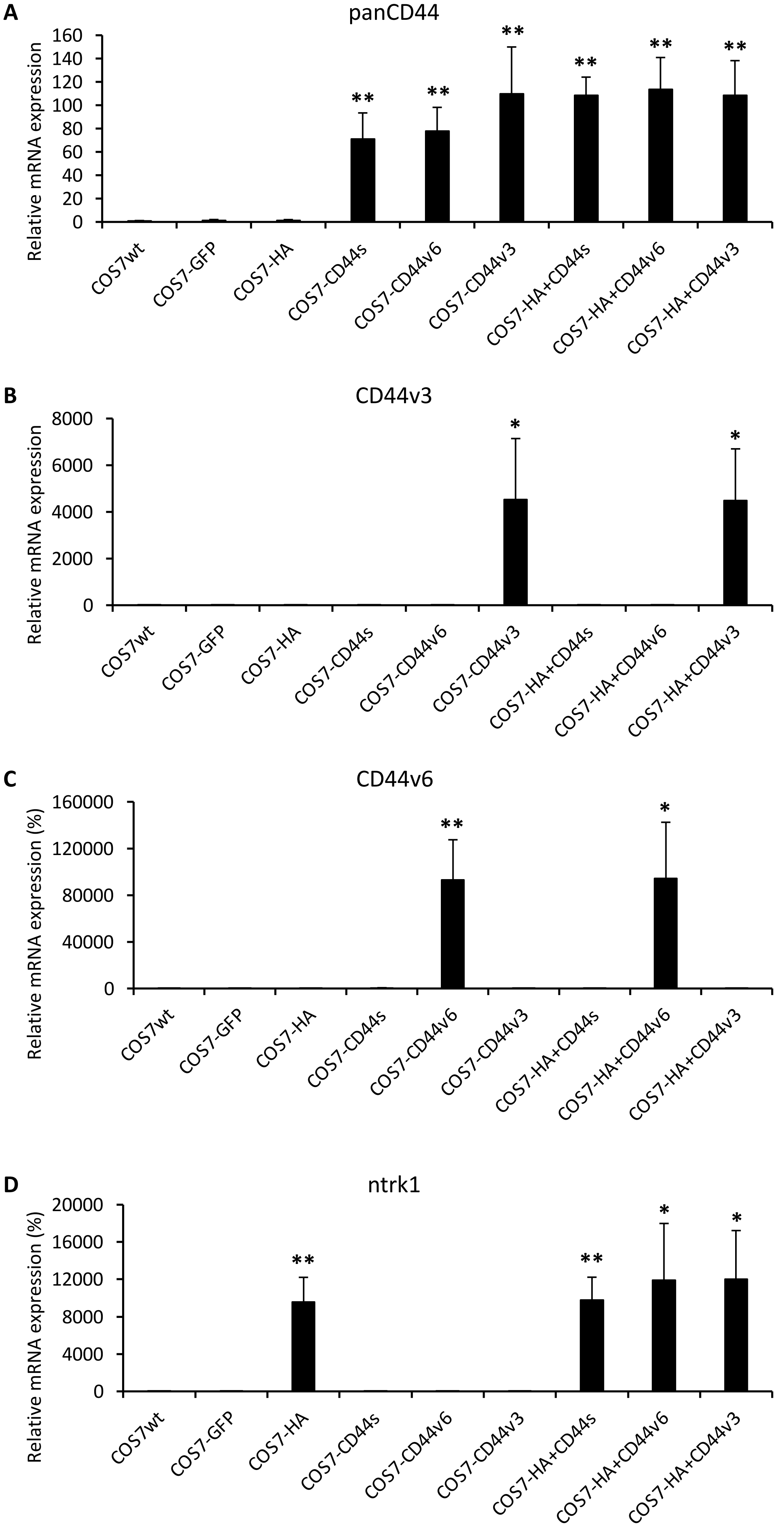

Figure Supp 1, Trouvilliez *et al.*

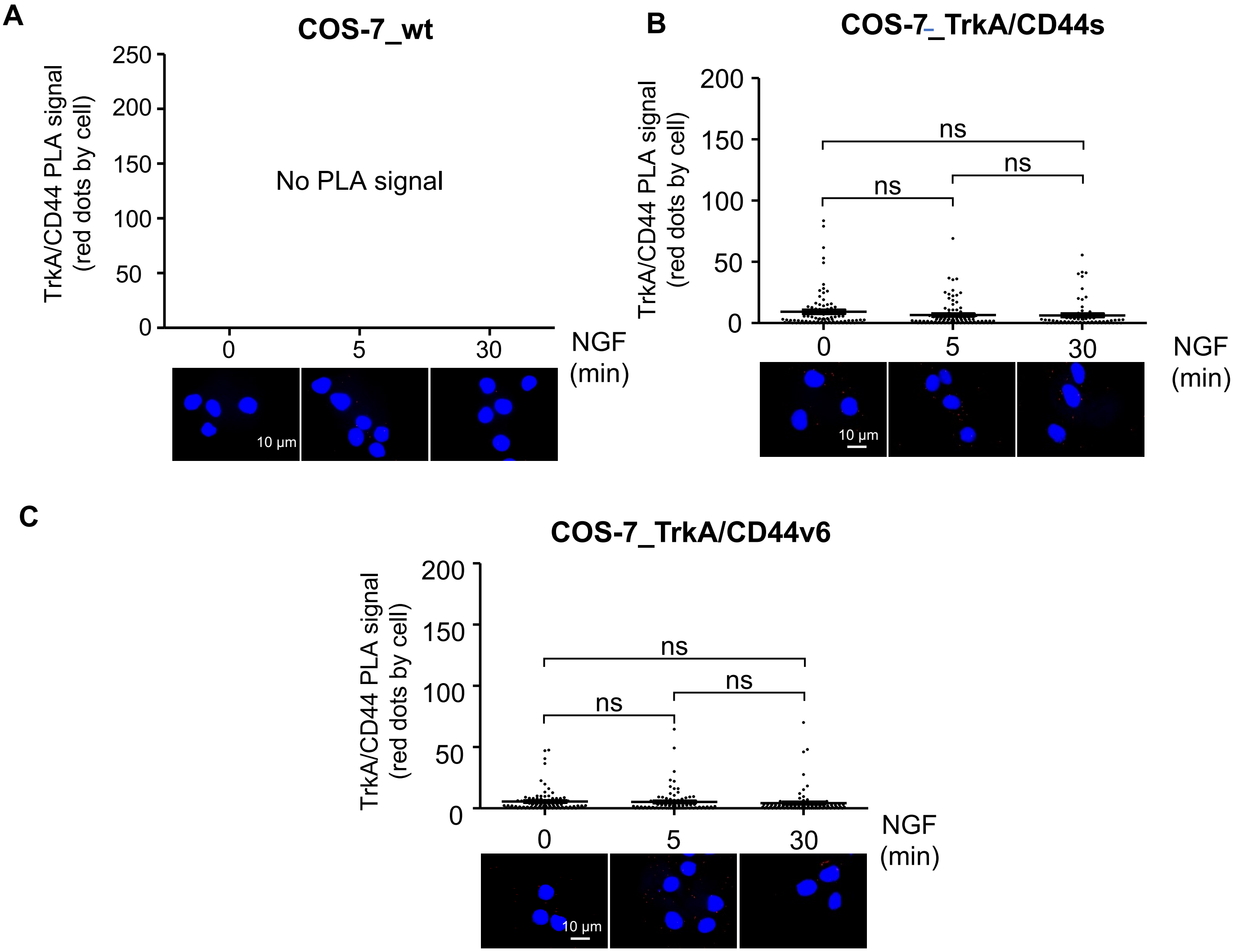

Figure Supp 2, Trouvilliez *et al.*

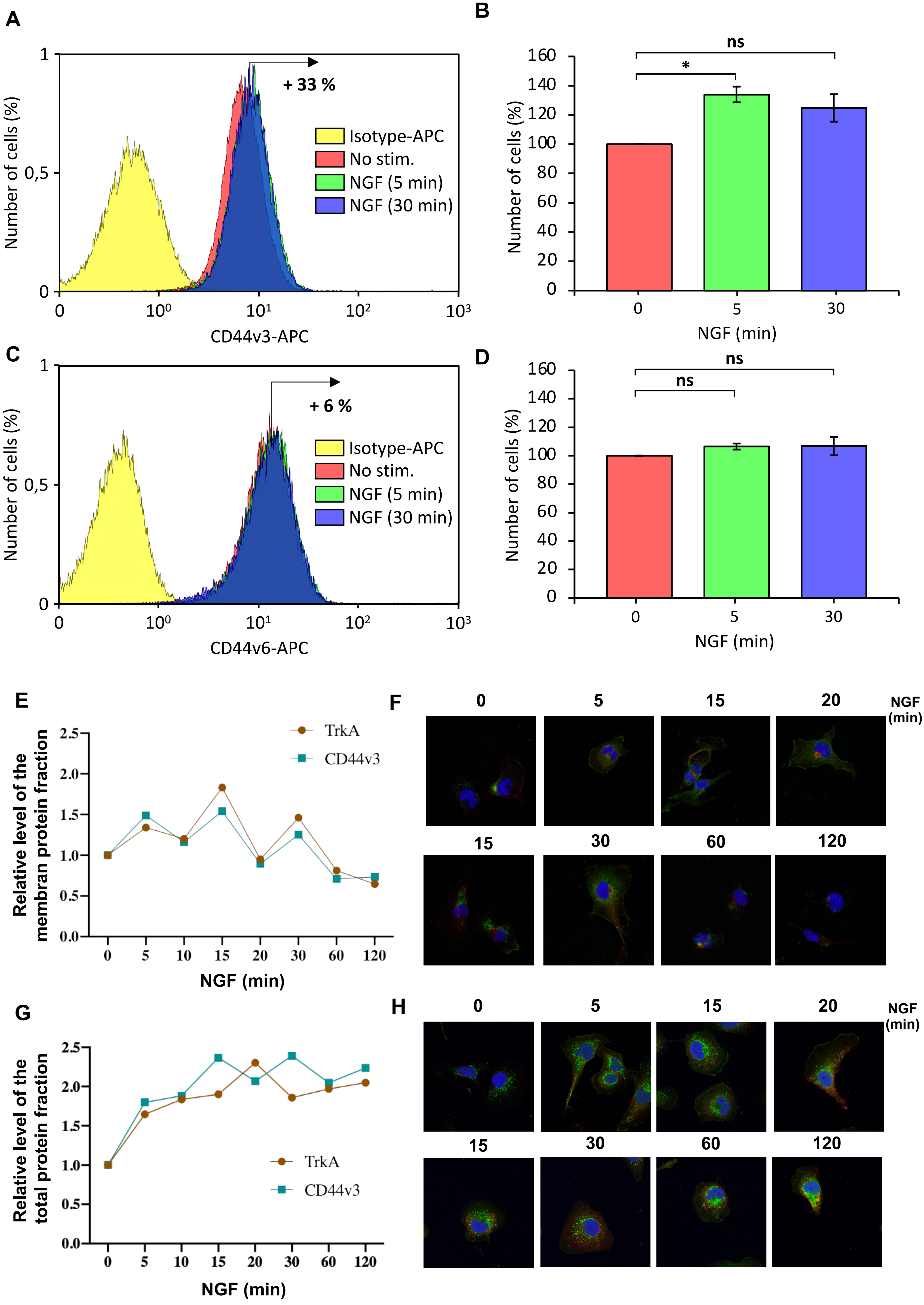

Figure Supp 3, Trouvilliez *et al.*

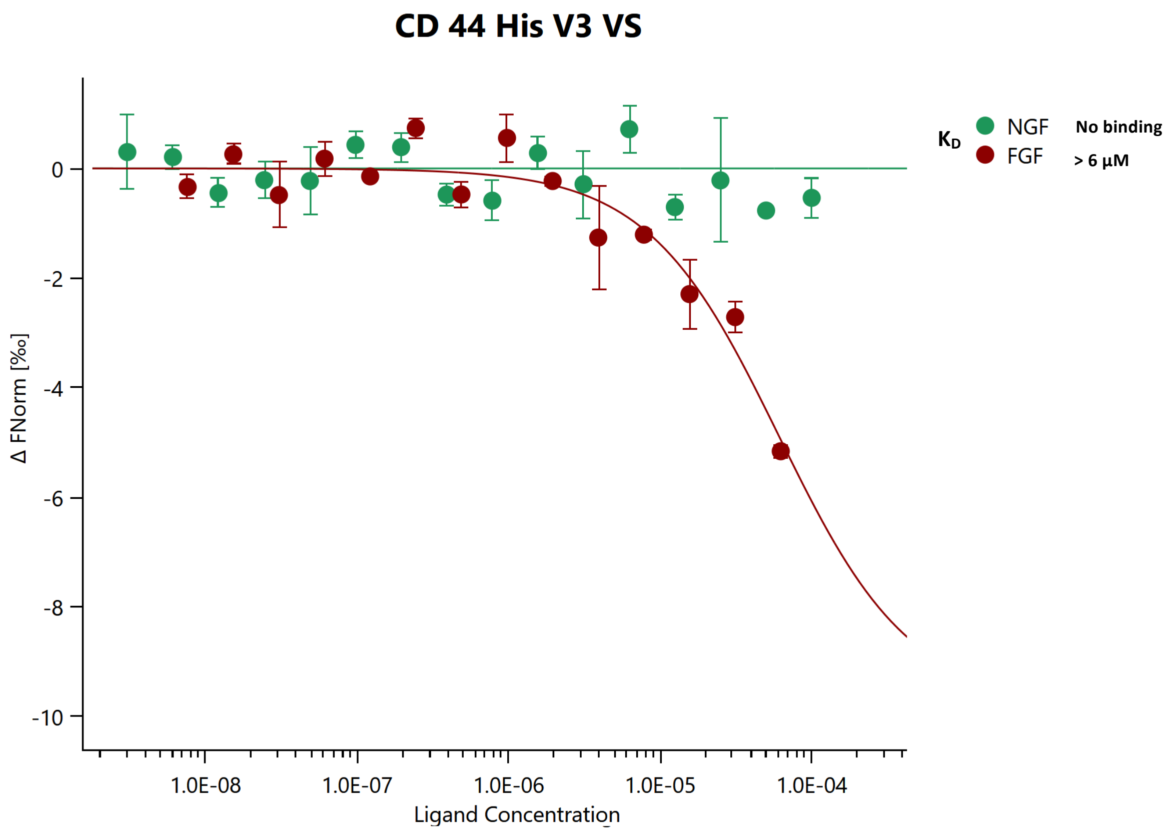

Figure Supp 4, Trouvilliez *et al.*

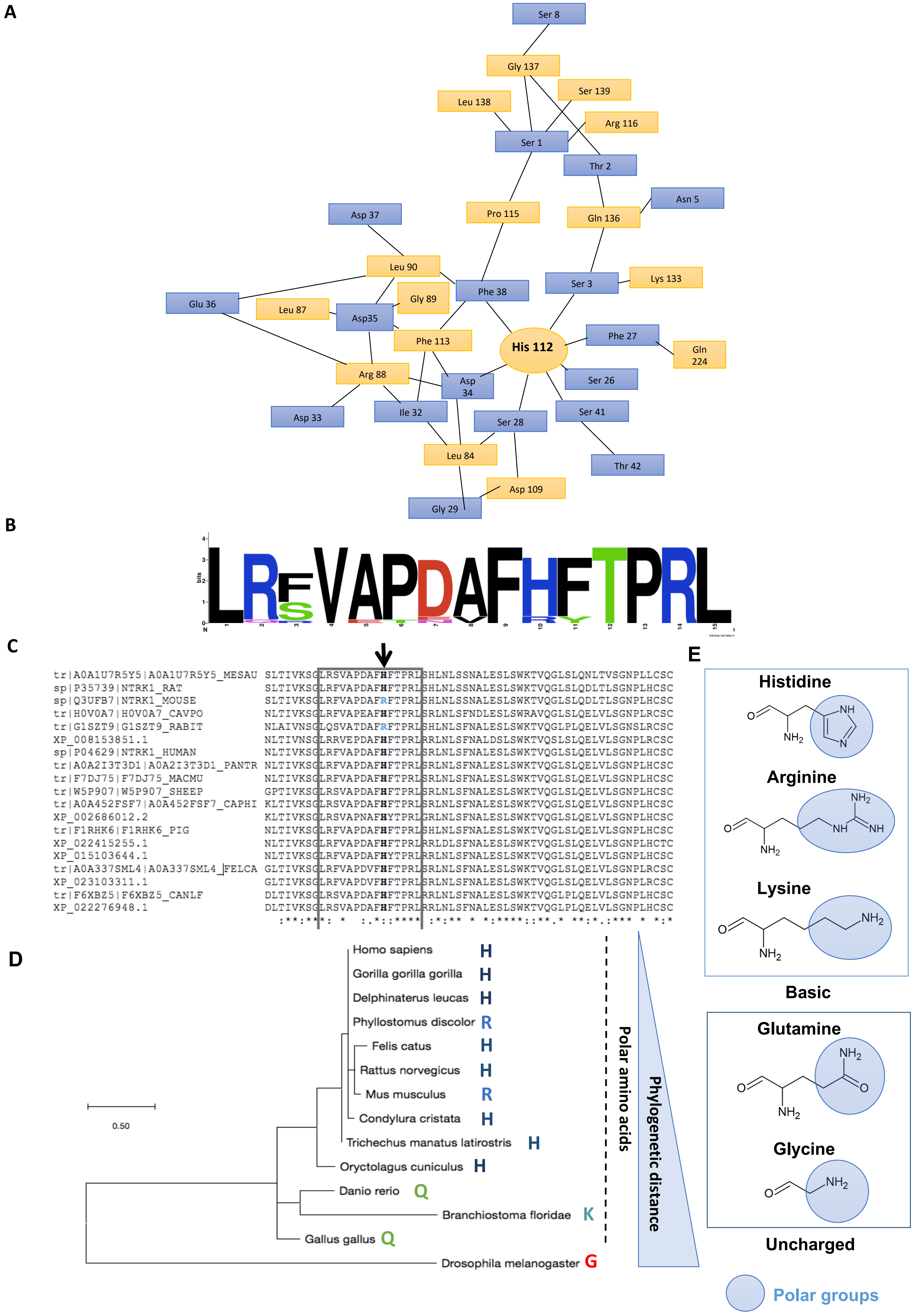
A
Ser 8
Gly 137
Ser 139
Leu 138
Arg 116
Ser 1
Thr 2
Asn 5
Pro 115
Gln 136
Asp 37
Leu 90
Lys 133
Phe 38
Ser 3
Gly 89
Glu 36
Leu 87
Asp35
Phe 113
His 112
Phe 27
Gln 224
Arg 88
Asp 34
Ser 26
Ile 32
Ser 41
Asp 33
Ser 28
Leu 84
Thr 42
Asp 109
Gly 29
B
bits
C
tr|A0A1U7R5Y5|A0A1U7R5Y5_MESAU SLTIVKSGLRSVAPDAFHFTPRLSHLNLSSNALESLSWKTVQGLSLQNLTVSGNPLLCSC
sp|P35739|NTRK1_RAT SLTIVKSGLRFVAPDAFHFTPRLSHLNLSSNALESLSWKTVQGLSLQDLTLSGNPLHCSC
sp|Q3UFB7|NTRK1_MOUSE SLTIVKSGLRFVAPDAFRFTPRLSHLNLSSNALESLSWKTVQGLSLQDLTLSGNPLHCSC
tr|H0V0A7|H0V0A7_CAVPO NLTIVKSGLRFVAPEAFHFTPRLSRLNLSFNDLESLSWRAVQGLSLQELVLSGNPLRCSC
tr|G1SZT9|G1SZT9_RABIT NLAIVNSGLQSVATDAFRFTPRLSHLNLSFNALESLSWKTVQGLPLQELVLSGNSLRCSC
XP_008153851.1 NLTIVNSGLRRVEPDAFHFTPRLRRLNLSFNALDSLSWKTVQSLSLQELVLSGNPLRCSC
sp|P04629|NTRK1_HUMAN NLTIVKSGLRFVAPDAFHFTPRLSRLNLSFNALESLSWKTVQGLSLQELVLSGNPLHCSC
tr|A0A2I3T3D1|A0A2I3T3D1_PANTR NLTIVKSGLRFVAPDAFHFTPRLSRLNLSFNALESLSWKTVQGLSLQELVLSGNPLHCSC
tr|F7DJ75|F7DJ75_MACMU NLTIVKSGLRFVAPDAFHFTPRLSRLNLSFNALESLSWKTVQGLSLQELVLSGNPLHCSC
tr|W5P907|W5P907_SHEEP GPTIVKSGLRSVAPDAFHFTPRLSRLNLSFNALESLSWKTVQGLSLQELVLSGNPLHCSC
tr|A0A452FSF7|A0A452FSF7_CAPHI KLTIVKSGLRSVAPDAFHFTPRLSRLNLSFNALESLSWKTVQGLSLQELVLSGNPLHCSC
XP_002686012.2 KLTIVKSGLRSVAPNAFHYTPRLGRLNLSFNALESLSWKTVQGLSLQELVLSGNPLHCSC
tr|F1RHK6|F1RHK6_PIG NLTIVKSGLRFVAPDAFHFTPRLSHLNLSFNALESLSWKTVQGLSLQELVLSGNPLHCSC
XP_022415255.1 NLTIVKSGLRFVAPDAFHFTPRLRRLDLSFNALESLSWKTVQGLSLQELVLSGNPLHCTC
XP_015103644.1 NLTIVKSGLRFVAPDAFHYTPRLRRLNLSFNALESLSWKTVQGLSLQELVLSGNPLHCSC
tr|A0A337SML4|A0A337SML4_FELCA GLTIVKSGLRFVAPDVFHFTPRLSRLNLSFNALESLSWKTVQGLSLQELVLSGNPLRCSC
XP_023103311.1 GLTIVKSGLRFVAPDVFHFTPRLSRLNLSFNALESLSWKTVQGLSLQELVLSGNPLRCSC
tr|F6XBZ5|F6XBZ5_CANLF DLTIVKSGLRSVAPDAFHFTPRLRRLNLSFNALESLSWKTVQGLPLQELVLSGNPLHCSC
XP_022276948.1 DLTIVKSGLRSVAPDAFHFTPRLRRLNLSFNALESLSWKTVQGLPLQELVLSGNPLHCSC
D
Homo sapiens H
Gorilla gorilla gorilla H
Delphinaterus leucas H
Phyllostomus discolor R
Felis catus H
Rattus norvegicus H
Mus musculus R
Condylura cristata H
Trichechus manatus latirostris H
Oryctolagus cuniculus H
Danio rerio Q
Branchiostoma floridae K
Gallus gallus Q
Drosophila melanogaster G
0.50
Polar amino acids
Phylogenetic distance
E
Histidine
Arginine
Lysine
Basic
Glutamine
Glycine
Uncharged
Polar groups

A **TrkA + SP**

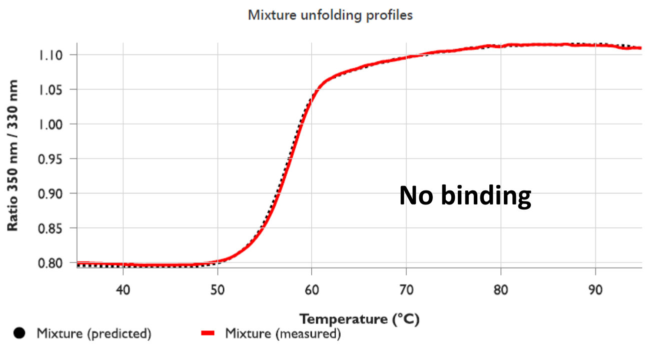

B **TrkA + P4**

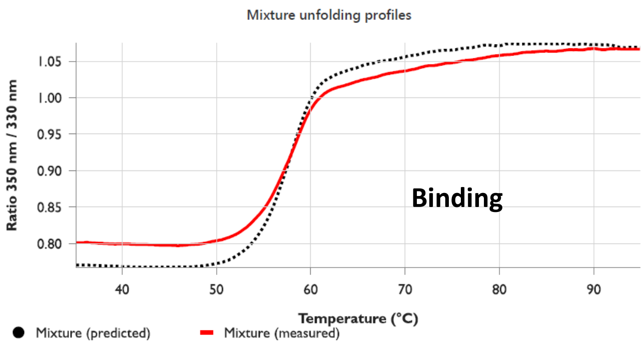

C **COS-7 CD44v3 + P4**

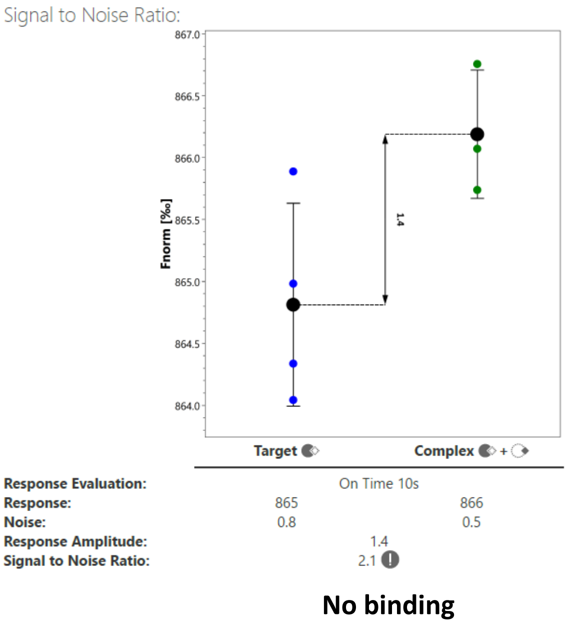

Figure Supp 6, Trouvilliez *et al.*

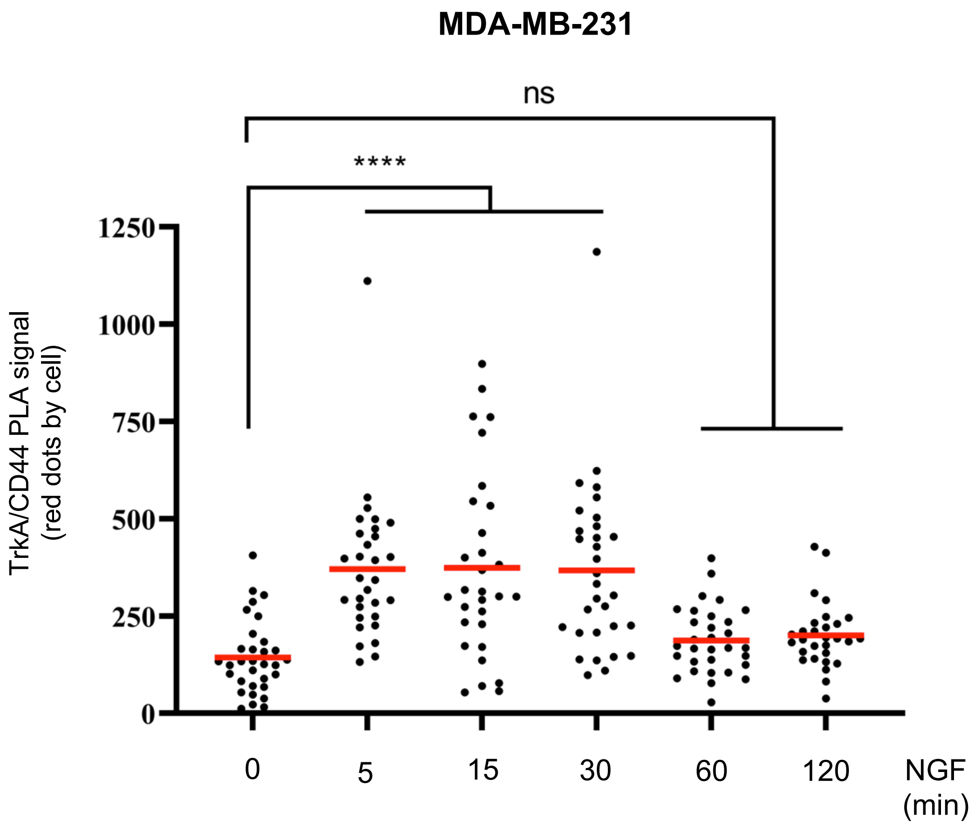

Figure Supp 7, Trouvilliez *et al.*

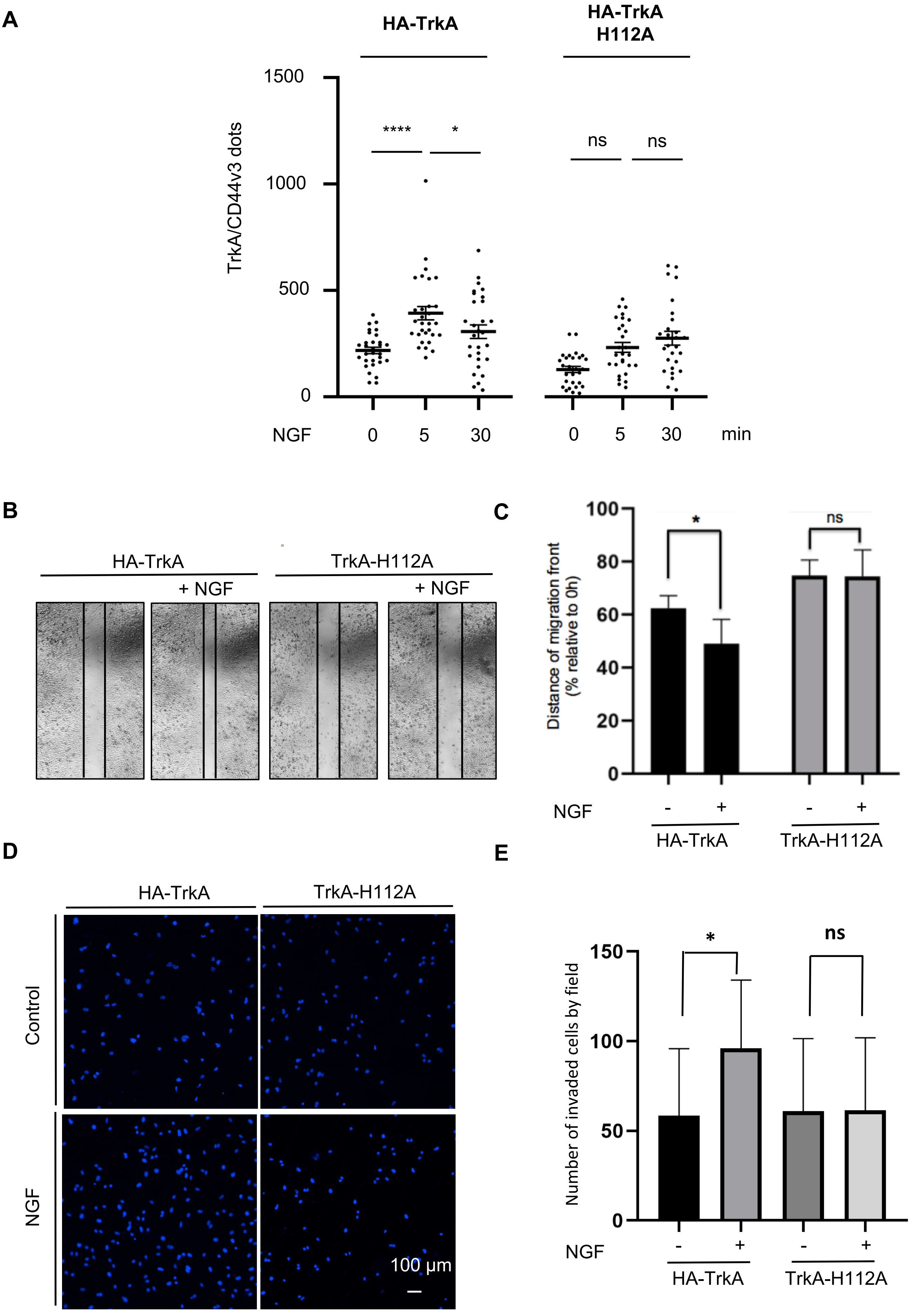

Figure Supp 8, Trouvilliez *et al.*

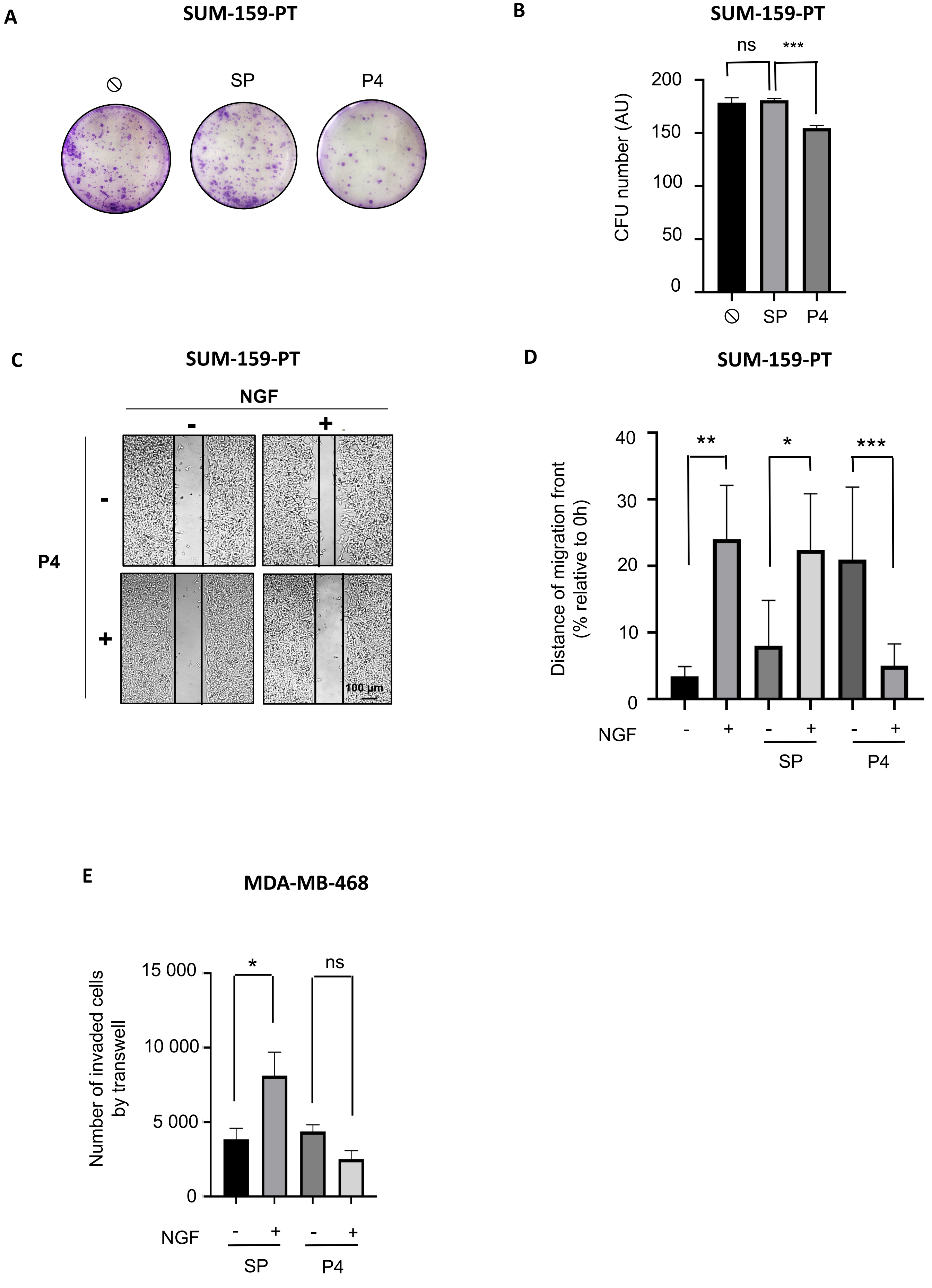

Figure Supp 9, Trouvilliez *et al.*

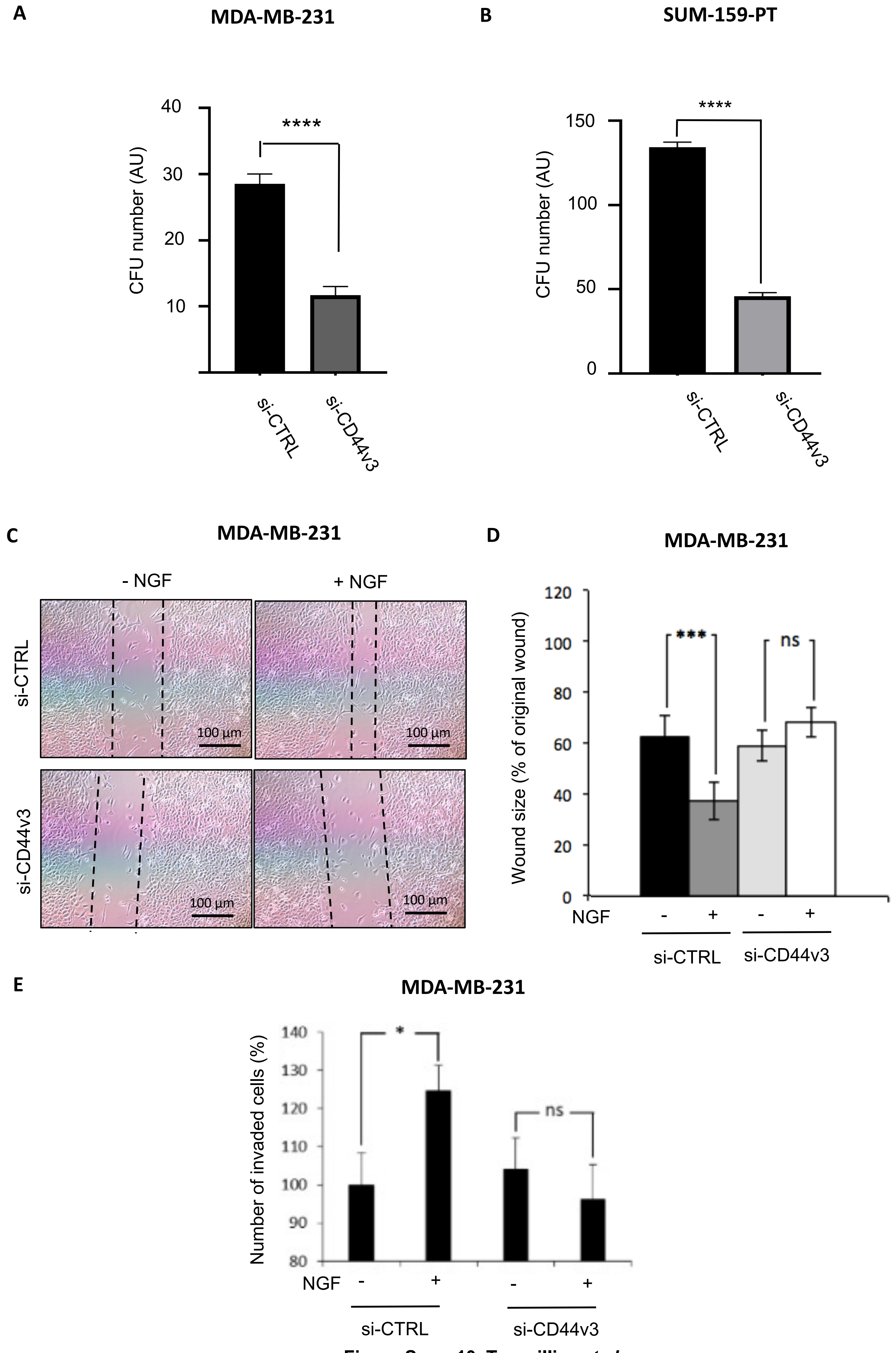

Figure Supp 10, Trouvilliez *et al.*

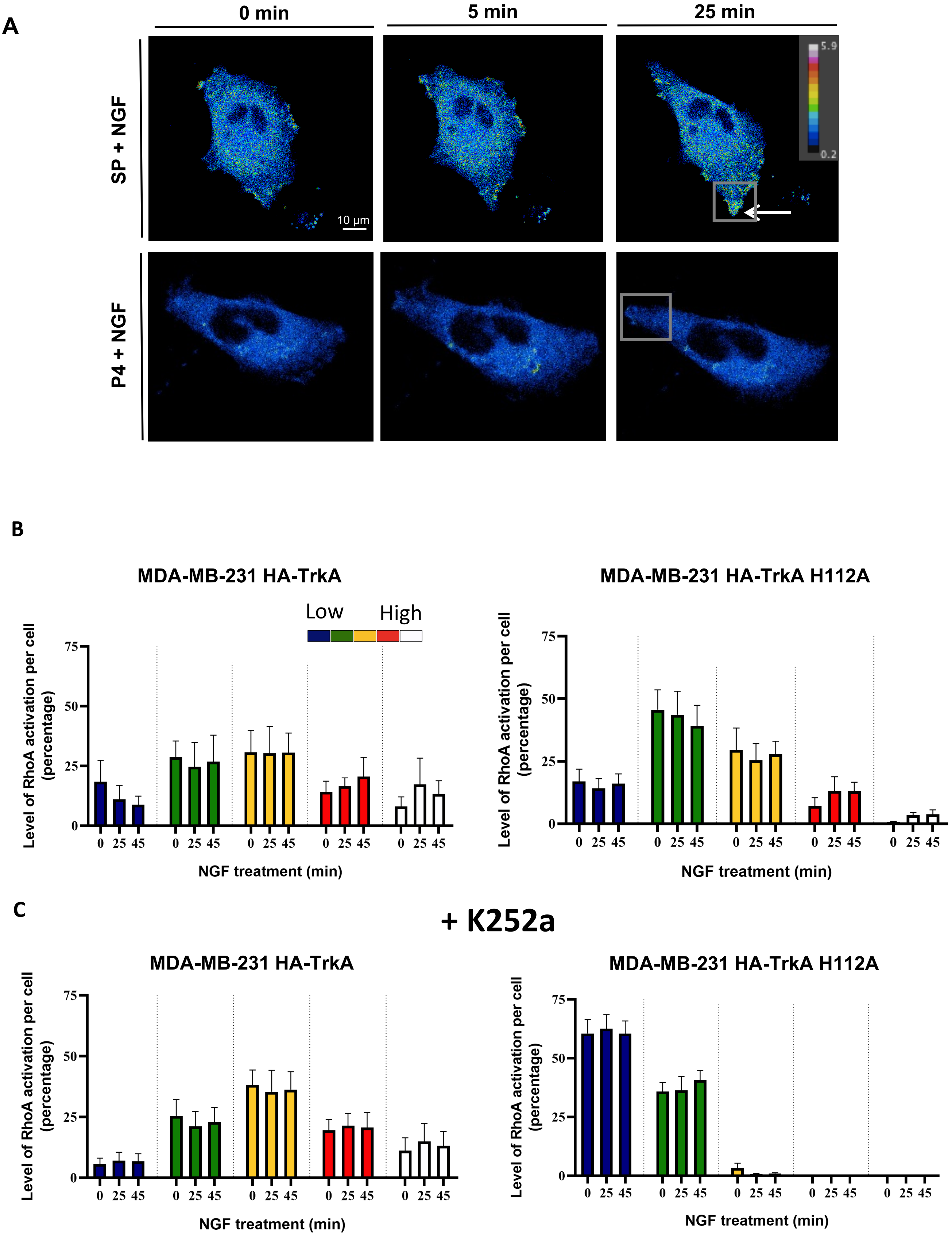

Figure Supp 11, Trouvilliez *et al.*
